# Supplementary material for: Overview of the Development, Impacts, and Challenges of Live-Attenuated Oral Rotavirus Vaccines
Source: Vaccines (Basel). 2020 Jun 27;8(3):341. doi: 10.3390/vaccines8030341 (PMC7565912; doi:10.3390/vaccines8030341)
Supplement: Supplementary file 1 [file vaccines-08-00341-s001.pdf]

Supplementary

Table S1. Efficacy and safety trials of the early rotavirus vaccine variants derived from animal and human rotavirus strains.

Vaccine candidate/variant	*Target age*	*Findings*
**Bovine rotavirus (BRV) vaccine variants**		
RIT4237 L991/$10^{8.1}$ $TCID_{50}$/dose	Finland (5 – 6 months)	Pre-inoculation seropositive children showed no serological response due to early exposure/maternal antibody. Seroconversion rate was 88% (breast-fed) vs. 45% (control). No major side effect observed except a mild RVD. Vaccine infectivity was suggested to be weakened by the gastric acidity [1-3].
RIT4237 at $10^{8.3}$ (Lot L1109), $10^{7.2}$ (Lot L993), and $10^{6.3}$ (Lot L1120) $TCID_{50}$/dose	Finland (4 – 6 months)	Vaccine efficacy examined in the breast-fed, formula-fed, and unfed children. Efficacy was correlated with vaccine doses with 100% seroconversion rate in the formula-fed. Response in the breast-fed was also remarkable. Hence RIT4327 is a potential rotavirus vaccine candidate [4].
2Ds each of RIT4237 from Lot L1139 ($10^{8.3}$ $TCID_{50}$) and L1189 ($10^{7.3}$ $TCID_{50}$)	Finland (6 – 12 months)	The serum anti-rotavirus antibody was similar irrespective of the feeding patters (breast-fed vs. bottle-fed); however, a marginal increased in seropositive was observed with higher vaccine dose (78% vs. 59%) [5].
RIT4327 at $10^{8.3}$ (Lot L1109)	Finland (neonates)	There was no significant protection offered by this vaccine in the neonates after 16 months of follow-up. There was no clear correlation between the vaccine-induced clinical protection and initial serological response [6], though, this vaccine modified the severity of RVGE.
1D each of RIT4237 (10^{7} $TCID_{50}$/mL) and (10^{8} $TCID_{50}$/mL)	USA (at 3 and 5 months)	At six months, cumulative homotypic seroconversion rate was 87% [7]. This trial failed to yield similar results in the developing countries of Latin America and Africa, hence withdrawn from the human trial [8].
3Ds RIT4237 with OPV	Gambia (≥ 10 weeks)	High prevalence of pre-vaccination rotavirus neutralising antibody. The overall vaccine efficacy of 33% with 92% of infants developing clinical rotavirus infection [9]
2Ds RIT4237 without OPV	Rwanda (at 3 and 8 months)	There was no significant difference in the episodes of diarrhoea, rotavirus shedding, and seroconversion rate among the vaccine and the placebo groups [10].
**Rhesus rotavirus (RRV) vaccine variants**		
Simian RRV (>10^{4} PFU/mL/dose)	<12 months	A reactogenic vaccine candidate in neonates and infants [11,12], which was normally antagonised by the maternal anti-rotavirus antibody. Nevertheless, it induced higher homotypic protection than the bovine rotavirus (BRV) vaccine candidate. Its VP7 was very similar to HRV serotype 3 unlike BRV [13,14].
RRV MMU18006 (**Lot RRV-1**) at 10^{5} FFU/mL	Venezuelan (5 – 20 months)	High fever (≥38.3°C) occurred 3 – 4 days post-vaccination. This vaccine was 100% infective and immunogenic without any serious adverse events. There was no evidence of viral transmission either by shedding or seroconversion [12].
Phase I trial with RRV at ≈$10^{6.5}$ PFU/mL	8 – 61 months	Well-tolerated with >4-fold increase in serum antibody but characterised with rhinorrhoea and high viral shedding [15]. Phase II trial with 1:10 vaccine dilution induced 100% 4-fold increase in serum antibody, no fever, no diarrhoea but rhinorrhoea and viral shedding persisted [15].
RRV-1 at 10^{6} $TCID_{50}$/mL	3 – 144 months	High pre-existing anti-rotavirus antibody. Well-tolerated but with fever. Highly immunogenic in 82% vaccinees with induced enteric and humoral immunity between 3 – 4 days post-vaccination, especially in the children with prior exposure to RVI [16].

Vaccine candidate/variant	***Target age***	***Findings***
RRV-1 at 10^5 PFU/mL/dose from 1:10 vaccine dilution	USA (4 – 24 months)	With feeding being withheld, the overall vaccine efficacy was 74% against all forms of diarrhoea. High vaccine "take", no viral shedding, highly immunogenic, protective, but with a proportion of febrile fever [17].
RRV-1 at 10^4 PFU/mL/dose	USA (2 – 4 months)	The vaccine was well-tolerated vaccine but with significantly high febrile fever and loose stools. About 52% breast-fed and 73% formulated infants developed ≥4-fold serum neutralising antibody. Though highly immunogenic yet rotavirus-associated infection was similar in the vaccinated and placebo groups purposely because the vaccine failed against the circulating serotype 1 (Wa) in Rochester. RRV-1 induced 4.7% heterotypic protection against serotype 1 vs. 58% homotypic protection against serotype 3 [18]. A need for polyvalent vaccine candidate was therefore suggested.
1D RRV-1 at 10^4 or 10^3 PFU/mL	3 – 11 months	These vaccine doses induced fever. Higher dose correlated with higher seropositivity. Pre-vaccination neutralisation antibody in infants <5 months limited the fever and favoured higher immunogenicity and safety of 10^4 PFU/mL vaccine dose [11].
RRV-1 in three different doses – 10^5, 10^4 and 10^3 PFU/mL	3 – 20 months	Homotypic seroconversion to serotype 3 was in 91% children while 14% displayed heterotypic seroconversion to serotypes 1 or 2 [19]. At least 88% of the children receiving any of vaccine doses had one class of rotavirus-specific serum antibody – IgG, IgA, and IgM, with the highest response from IgA (69%) [19], which was also found in the stools – meaning that mucosal secreted IgA may be correlated to protection against RVI in children [20].
Phase I trial of 10^4 and 10^3 PFU/mL/dose of RRV-1 from 1:100 and 1:1000 dilution of 10^6 PFU/mL	Venezuelan (1 – 10 months)	Immunogenicity correlated with vaccine dose. It was non-reactogenic. Viral shedding increased with dose but decreased with age. The 4-fold increase antibody seroresponse to RRV-1 was 59% (10^3 PFU/mL) and 82% (10^4 PFU/mL) [21]. The 10^4 PFU/mL used in the Phase II induced only high fever >39°C with seroresponse rate of 69.3% in 4 – 10 months age group vs. 75.5% in the 1 – 4 months age group. High pre-existing anti-RRV1 neutralisation antibody was reported, which must have improved the IgG and IgM seroconversion [21]. A similar observation was later observed and reported in Finland when RRV-1 was tested in infant 2 – 5 months of age [22].
RRV-1 at 10^4 PFU/mL/dose	Venezuelan (1 – 10 months)	Overall vaccine efficacy of 68% was observed against any RVD with evidence of clinical severity and age-dependent efficacy; 93% in 1 – 5 months age group and 100% protection against the most severe rotavirus diarrhoea (SRVD) [23]. RRV-1 displayed significant heterotypic protection against subgroup II rotaviruses - including serotypes 1, 3, and 4 [24].
RRV-1	Adults	There was a considerable level of heterotypic protection against the HRV strains Wa and DS-1, which was absent in children [19]. This showed that pre-existing RVI improves heterotypic protection in adults. With characterised serotype-specific monoclonal antibody against the VP7, a competitive solid-phase immunoassay to measure the epitope-specific immune responses in children that received RRV-1 (serotype 3) vaccine showed only 11% sera sample with seropositive to serotypes 1 and 2 while 72% of the sera sample was seropositive to serotype 3 in children [25]. Though this work showed huge homotypic protection against serotype 3, considerable heterotypic protection was observed. Furthermore, VP4-specific monoclonal antibody, which had been shown to bind heterotypic neutralising domains of serotypes 3, 5, and 6 [25] but not serotypes 1 and 2 [19], was used to detect the presence of similar antibodies in 56% of children sera immunised with RRV-1. This further confirmed that RRV-1 could induce a detectable response to VP4, which mediated heterotypic immune response to serotypes 3, 5, and 6. This means antibody response to the heterotypic neutralising domain of VP4 needed considerable insight. Though its seropositive response was smaller compared to the VP7 immune response, but its

Vaccine candidate/variant	*Target age*	*Findings*
		contribution to the heterotypic protection of rotavirus vaccines cannot be ruled out. Besides, multiple natural infections with serotype 3 have been linked to homotypic and heterotypic protection against serotypes 1, 3, and 4 [26].
Phase I trial of RRV-1 at $10^{6.8}$ PFU/mL/dose at 1:10 of original vaccine dose	Sweden (4 – 12 months)	Yet, the occurrence of high fever >38°C and loose stools. Higher dilution (1:100) vaccine dose was less reactogenic in 2 – 5 months infants, associated with fever in >5 months old infants, and slightly less immunogenic response. The administration of vaccine buffer was also recommended.<br>The Phase II trial witnessed 92% seroconversion rate. The overall vaccine efficacy was 48%, accompanied by some mild and short duration reactogenic responses [27].
RRV-1	Finland (2 – 5 months)	About 26% developed febrile reactions, 62% with ≥4-fold serum neutralising antibody despite a high level of maternal anti-rotavirus antibody. The overall vaccine efficacy of 38% against RVD and 67% against severe-moderately severe RVD [22]. No evidence of prevention in the second year of follow-up but only that the vaccine modified the clinical severity associated with RVI [22]. Though RRV-1 is generally administered at 10^4 PFU/mL/dose to minimise reactogenicity, this was claimed as an insufficient dose that only induced partial heterotypic protection [18]. Therefore, heterologous vaccine candidate such as human-rhesus rotavirus reassortant was suggested to induce better heterotypic protections against HRV strains.
**Attenuated BRV vaccine candidate**		
WC3 at $10^{7.5}$ PFU/mL	5 months – 6 years	This demonstrated an efficient induction of serum neutralising antibodies without any adverse events. Evidence of seropositive antibody against more than one serotypes increased by age [28-30]. Homotypic protection was more prominent in the infants, but the heterotypic immune response was significantly higher in aged children. The viral shedding was about 30%.
1D of WC3 at 10^7 PFU/mL/dose adapted from serotype G6	USA (<12 months)	This induced 97 – 100% protection against SRVD caused by rotavirus serotype 1 but 9% seroresponse to other HRV serotypes [31,32]. It may boost the pre-existing naturally acquired protection against HRV serotypes G1 – G4 [8,32,33]. However, this vaccine candidate performed poorly with <48% protection in other regions, including the LICs against the SRVD [31].
**Human rotavirus (HRV) vaccine variants**		
RV3 serotype G3P2A	3 months	This induced neutralising antibodies to human serotypes G1, G3, and G4, which persisted and protected against clinical disease due to rotaviruses of G2 type during their first three years of life [8]. However, homotypic infection with RV3 may not confer immunity against rotavirus re-infection but can offer protection against clinical severity during re-infection [34].
Phase I trial with 1D of RV3 at 6.5 x 10^5 FFU/mL	Australia 21 – 30 years, 3 – 4 years and 3 months	There was evidence of mucosal and serum IgA antibody responses to G3 without any side effects and viral shedding [35]. Vaccine tolerance was age-dependent. No evidence of seroconversion to RV3, but a trace of immune response/serum neutralising antibody to RV3 with a slight heterotypic neutralising antibody against RV4 (G1 serotype) in infant group only [35,36].<br>Three doses Phase II trial showed similar results. There was a 46% overall immune response in the vaccinated infants. Soy-meal had no significant effect on the vaccine response. Furthermore, this vaccine induced twice the copro-IgA in the vaccinated infants as compared to the placebo but high prevalence of maternal anti-rotavirus IgG mitigated the efficacy of this vaccine candidate. The protective efficacy of the vaccine was 54% (immune-responders) vs. 55% (non-responders) vs. 53% (placebo) at the end of the first season. More vaccinated children developed episodes of diarrhoea after the two seasons of RVI caused the serotypes I (G2P1B[4]) and II (G1P1A[8]) with similar severity with the placebo [37], which means the

Vaccine candidate/variant	*Target age*	*Findings*
		vaccine failed to offer heterotypic protection beyond the first year [34], however, traces of protection against the predominant heterotypic rotavirus strain was described.
Cold adapted HRV IGV-80-3		This was passaged from 37°C to 25°C with remarkable immunogenicity in piglets and mice [38] (p. 315). It contains serotype 1 and subgroup II conserved antigens – VP4 and VP7 [39,40]. Other HRV cold-adapted vaccine variants include strain D (containing VP4: 1A; VP7: 1), DS-1 (containing VP4: 1B; VP7: 2), and human-human rotavirus reassortants like Wa x DS-1 (containing VP4: 1A; VP7: 2) and Wa x P (containing VP4: 1A; VP7: 3) [40]. These phenotypic strain vaccine candidates, which are cold-adapted (*ca*) and temperature-sensitive (*ts*), have been shown to induce broad cross-reactive neutralising antibodies in animal models [40] as well as in humans [41].
**Reassortant vaccine candidates**		
Human VP7 serotype G1 D x RRV at 10^4 PFU/mL and VP7 serotype G2 DS1 x RRV at 10^5 PFU/mL	Finland (2 – 5 months)	Reactogenic effects and adverse events were the same as the parent strains. However, 61% and 75% seroconversion rate were observed with serotypes G1 and G2, respectively. The two vaccines showed an equal successive reduction of clinical efficacy against G1 (92% - first and 59% - second epidemic seasons). Efficacy against asymptomatic RVI drastically reduced from 59% in the first season to zero in the second season [42]. Higher vaccine titre of G2 reassortant, however, showed significant heterotypic protection in Finland but failed to protect Peruvian children [8].
D x RRV and DS1 x RRV	Venezuelan (1 – 5 months)	Similar systemic reactions between the parent strain RRV and the reassortants were observed. High vaccine "take" (87 – 100%) and vaccine shedding (53 – 86%) were reported. Similar overall homotypic and heterotypic seroresponses to Wa, DS1, and RRV were similar. The significant role of VP4 in the development of neutralising responses in children after RVI was speculated. Seroresponse was indirectly related to the pre-existing anti-rotavirus antibody but directly related to the age group [43]. Serological IgA measured ranged from 57 – 88%.
3Ds of quadrivalent reassortant vaccine – RRV serotype 3 VP7 and three human-RRV reassortant strains (D x RRV (serotype 1 [VP7]), DS1 x RRV (serotype 2 [VP7]), and ST3 x RRV (serotype 4 [VP7])	Venezuelan (10 – 20 weeks)	Febrile reaction and high vaccine shedding were observed in 2.5 – 5 months children [44], which was similar to D x RRV and DS1 x RRV reassortant vaccine trial [43]. A range of 73 – 79% of infants who received any of the vaccine dose developed ≥4-fold increase neutralising antibody against RRV only. However, 4 – 23% from the low dose, 21 – 33% from the medium dose, and 32 – 58% in the high dose groups developed such response to HRV prototype strains Wa (serotype 1), DS1, P (serotype 3), and ST3 antigens [44]. Further analysis showed that the VP4 antigen from RRV component of the quadrivalent vaccine appeared more immunogenic than the VP7 from the human strain. Though seroresponses increased with increased vaccine dose, yet the response was significantly higher when such vaccine strains were administered individually.

D = dose; **BRV** = bovine rotavirus; **Ig** = immunoglobulin; **PFU** = plague forming unit; **RRV** = rhesus rotavirus; **RVD** = rotavirus diarrhoea; **RVGE** = rotavirus gastroenteritis; **TCID** = tissue culture infectivity dose.

Table S2. Vaccine coverage and overall effectiveness/efficacy of live-attenuated oral rotavirus vaccines, Rotarix® and RotaTeq®.

Country	*Coverage/ Surveillance*	*Vaccine Coverage*	*Associated vaccine impacts*
**Europe**			
Austria	2010 – 11<br>2007 – 08	78 – 84%<br>72 – 87%	95 – 96% VE respectively for the full course of a vaccine against RVGE hospitalisations [45]<br>Overall field effectiveness between 61 – 98% [46]. Generally, RoV introduction has reduced hospitalisation rate and nosocomial GE due to RVI in children of all ages with sustainable protection over more than three years [45,47,48]
Belgium	2007 – 09	85 – 90%	Efficacy of 91% for ≥1D, overall of 90% against RVGE, 91% against SRVGE, and 66% against mild-moderate RVI [49][49][49][49][49]. With this introduction, all seasonal rotavirus-associated hospitalisations reduced [50,51] including paediatric rotavirus infections in the first and second year after vaccination [52] and all laboratory-confirmed cases [53]
Finland	2010 – 12	95 – 97%	Effectiveness of 92.1% and 78% against RVGE hospitalisations with RV5 over three years of prospective monitoring among eligible children and all children <16 years, respectively [54]. When the pre-vaccine period was compared with the post-vaccine period, an efficacy of 80.3% for the hospitalised inpatient and 53.9% for the total inpatient with RVGE hospitalisation in <1-year toddlers were observed. The vaccine further produced efficacy of 78.8% for the outpatient and 12.5% for the total outpatient with AGE burden, and the overall efficacy of 97% against confirmed cases of RVGE [55]. Similarly, after two years of National Immunisation Programme (NIP), RV5 reduced RVGE hospitalisation, outpatient clinic visit, all hospitalisation, and all outpatient clinic visit for GE of any cause by 76%, 81%, 57%, and 62% respectively [56]
France	2008 – 09	47.1%	A relative risk reduction of 98% in RAGE hospitalisation within two years of vaccine era [57]
Germany	2006 – 10	3.8 – 56%<br>2.5 – 28%	Substantial reduction in rotavirus-related hospitalisation though with relatively low coverage of 56% (average) and 28% (low) in the Eastern and Western Federal States coupled with immunisation completeness of 91% (in the Eastern Federal States) and 96% (in the Western Federal States). Compared with the pre-vaccine era, RoVs have brought a drastic reduction in the number of incidences of rotavirus-related hospitalisation and nosocomial RVIs in children <23 months of age within the first two years of life. This was accompanied by a very low incidence rate ration [58]<br><br>There was a report of the high occurrence of G1P[8] and G9P[8] in RV5-vaccinated children while G2P[4] and G9P[8] were common with RV1-vaccinated children between 2010 – 11 in a case-control risk factor study, however, 68% and 80% VE against RVI requiring medical attention and hospitalisation were reported for children aged 6 – 29 months. Other impacts include stable prevention and reduction over two years against hospitalisation and less hospitalisation in the vaccinated (23%) vs. unvaccinated (61%) children. Breastfeeding and day-care attendances were two independent risk factors against RVVE [59]
Moldova	2012 – 14	20 – 40%	With this overall vaccine coverage for all children <5 years old, hospital admission for rotavirus positivity fell from 45% in the pre-vaccine era to 25% in 2013 and 14% in 2014 with significant reduction among infants <1-year-old and herd protection among aged cohorts [60]
Portugal	2007 – 10	16 – 42%	A surveillance study between 2006 – 10 could not observe any significant trend in the efficacy of RoV purposely because of the low vaccine coverage. No progressive trend or seasonal shift in RVGE cases [61]. Surveillance study between 2006 – 12, however, showed a very high VE against RVGE hospital attendance and admission despite this low vaccine coverage. At least 1D of the vaccines produced 83.7% and 96.1% also FD vaccines produced 83% and 97.5% effectiveness against hospital attendance and admission, respectively. No hospital admission recorded after the FD of RV5 vaccine. There was no significant difference in the effectiveness of the two vaccines [62]

Country	*Coverage/ Surveillance*	*Vaccine Coverage*	*Associated vaccine impacts*
Spain	2006 – 09	12 – 51%	VE against any episode of RVGE was 91.5% and against hospitalised RVGE was 95.6% in <2 years old children that received ≥1D. In fully dosed children <2 years old, the effectiveness of 92.8% against any episode of RVGE and 98.3% against hospitalised RVGE were observed. Lastly, in partially dosed children <2 years old, 84% and 89.4% VE were recorded against any episode of RVGE and hospitalised RVGE respectively. There was no significant difference between the VE of RV1 and RV5 [63]. Castilla and co-workers in 2012 reported 78% VE against RVGE and 83% VE against hospitalised RVGE from complete vaccination programme [64]. The relatively low vaccine coverage in Spain showed a huge reduction in RVGE hospitalisation, all-cause diarrhoea, and diarrhoea of undetermined aetiology when pre-vaccine era (2005 – 06) was compared with the two consecutive years of vaccine assessment era (2008 – 09) in children <5 years old. The greater impact was observed in infants <1-year-old [65]. A similar reduction was observed when the pre-vaccination era (2003 – 07) was compared with the vaccination era (2008 – 10) [66]
**North America**			
Canada	2012 – 14<br>2013 – 14<br>2012 – 13<br>2011 – 12	46.4%<br>82.9%<br>35%<br>7.3%	With 91.4% of infants receiving only 1D or 2Ds of RV1, >90% VE was observed over 2 years [67]<br>By 2014, there was a relative decrease of 70.1% in rotavirus prevalence as well as 77% and 69% decrease in homotypic and partly heterotypic strains prevalence respectively, but the effect against the heterotypic was weak in children <3 years old (8% in 2012, 46% in 2013 and 23% in 2014) [67]
USA	2010 – 15<br>2007 – 09	59 – 73%<br>28 – 62%	Overall 91% VE for RV1 and 92% for RV5 against SRVGE [68]<br>Overall 70% VE for RV1 and 84% for RV5 against RVGE hospitalisation and emergency visit [69]<br>Overall 80% VE each for RV1 and RV5 against RVGE [70]<br>3Ds of RV5 produced 89 – 94% VE in ≥8 months old babies, 86 – 92% in ≥24 months old babies. 2Ds produced ≥90% VE in ≥8 months old babies and 1D with 66% VE in babies from 6 weeks to 5 months old [71]<br>Overall 74% VE for 1D, 88% for 2Ds and 87% for 3Ds of RV5 against RAGE hospitalisation and emergency visit [72]
**Central America**			
El Salvador	2007 – 09<br>2008 – 18	58 – 61.4%<br>81 – 99%	Reduction of all-cause diarrhoea mortality in <1-year-old babies was 47.6% and in <5 years old children was 49.5% with FDs of RV1 [73]<br>By the end of 2008 and 2009, RV1 had reduced the hospital admission rate for diarrhoea in children <5 years old by 40% and 51% respectively while achieving 76% VE for FDs of RV1 (1D produced 51% VE) against diarrhoea requiring hospitalisation between 2008 – 09 sentinel study [74]
Guatemala	2010 – 18	38 – 87%	Combining the effectiveness of 2 – 3Ds of RV1/RV5 against severe rotavirus-related diarrhoea with hospitalisation or emergency hospital visit between 2012 – 13, 73% was observed with the HC analysis while 49% was observed for the TNC analysis. For the VSRVGE, 76% and 44% were respectively observed for the HC and TNC analysis. There was no significant difference between the vaccine complete courses, RV1 (63%) vs. RV5 (69%). G12P[8] accounted for 89% of cases that showed partial heterotypic protection to both vaccine strains [75]
Mexico	2007 – 08<br>2007 – 09	74 – 89%<br>35.9 – 88.7%	Between 2007 – 08, about 56% reduction in diarrhoea-related mortality in <1-year and 46% in <5 years old children were observed [76]<br>In 2008 – 09, there was a reduction of 11 – 40% in diarrhoea-related hospitalisation in <5 years old children and the highest reduction occurred in children <1-year-old with 25 – 52% following 1D of RV1 [77]<br>With RV5, reduction of all-cause diarrhoea mortality in <1-year-old infants was 35.6% and <5 years old children is 33.9% [73]
Nicaragua	2006 – 07<br>2007 – 10	61 – 82%<br>26 – 86%	With 3Ds of RV5, 46% VE was observed against hospitalised RVI, 58% VE against SRVGE, and 77% against VSRVGE. Differential vaccine coverage was reported as followed: 98% for the 1D, 93% for the 2Ds, and 77% for the 3Ds [78,79]

Country	*Coverage/ Surveillance*	*Vaccine Coverage*	*Associated vaccine impacts*
	2007 – 09	11 – 59%<br>79 – 94.4%	With average vaccine coverage of 79% in babies <1-year-old and 35% in 1 to 4 years children, overall VE of 45% compared to the TNC group and 70% compared to the NDC group were observed for all ages against RVGE. Again, 51% VE was observed against SRVGE and 58% against VSRVGE. The overall risk of rotavirus hospitalisation was twofold lower in <1-year-old infants compared to ≥1-year-old children receiving RV5 [80]<br>With RV5, reduction of all-cause diarrhoea mortality in <1-year-old infants was 30.1% and <5 years old children was 36.4% [73]
[a]Panama	2007 – 09	68.4 – 77%	With RV1, reduction of all-cause of diarrhoea mortality in <1-year-old infants was 8.2% and <5 years old children was 3% [73]
**South America**			
Bolivia	2009 – 11	65 – 80%	59% overall VE in all ages with 2Ds of RV1 against all severity but 26% VE with 1D [81]. There is a significant impact of 2Ds over 1D against all severity in Bolivia<br>Additionally, in all ages with RV1 comparing to either NDC or TNC, overall VE of 77% or 69%, 76% or 69%, and 85% or 80% were respectively observed for all RVGE, SRVGE and VSRVGE [82]
Brazil	2007 – 09<br>2008 – 11<br>2006 – 11	76 – 81.9%<br>64.3 – 72.3%<br>46.5 – 87%	Reduction of all-cause diarrhoea mortality in <1-year-old infants was 45% and in <5 years old children was 42.1% with FDs of RV1 [73]<br>Overall VE of 75.8% compared to the NC group or 40% compared to the HC group against SRVGE with RV1 was observed [83-85]
Colombia	2011 – 13	94%	Overall VE of 79.19% against all severity of rotavirus diarrhoea with emergency hospital visit in 6 – 11 months old infants vaccinated with RV1 but drastically reduced to -39.85% in children >12 months old [86]<br>A previous report by De La Hoz and co-workers in 2010 stated 27% reduction in all causes of rotavirus-associated diarrhoea cases and 25% reduction in expected death rate associated with diarrhoea when vaccinated with RV1<br>About 30% reduction cases of rotavirus-associated diarrhoea and 28% reduction in the expected death rate from diarrhoea with RV5 when RoV immunisation was rolled out in 2009 for babies <2 years old have been recorded [87]. These observations with RV1 and RV5 showed no significant difference in the efficacy and protection offered by either of these vaccines in Colombian infants
**Asia**			
Armenia	2012 – 15	16 – 77%<br>81%	Within the first year of vaccine introduction, rotavirus hospitalisation reduced by 48% and more significantly by ≥75% in the second and third-year post-vaccination and compared to the pre-vaccination in the children <1 year. With 81% vaccine coverage in children ≥1-year, hospital admission due to RVI serially declined from 31% in 2013 to 82% in 2015. Additional herd protection was observed up to 48% in older children (2 to 4 years) by 2015 [88]
Bangladesh	2008 – 11	73.7 – 70.4%	VE against any severe and severe ARD was 29.0% and 22.9% after 6 weeks post-vaccination and 24.9% and 20.4% after 20 weeks post-vaccination respectively. The point estimate for total VE was higher against ARD in the first year than the second year of life (45.2% vs. 28.9%), and none of the fatal adverse events was associated with the vaccine intake [89]
**Middle East**			
Israel	2007 – 09	55%	With purchase rates of 92.1% for RV1 and 7.9% for RV5 reaching a vaccine uptake of 55.1% (mostly RV1) in the studied cohort, 50.1% VE was observed against hospitalised RAGE from 23.2% and 46.4% risk of AGE among the vaccinated and unvaccinated children respectively [90]. In another analysis, FD vaccination yielded 89.4% against RVGE hospitalisation, and any 2 – 3Ds of the vaccines produced 88.9% effectiveness [91]
**Africa**			

Country	*Coverage/ Surveillance*	*Vaccine Coverage*	*Associated vaccine impacts*
Botswana	2012 – 18<br>2013 – 18<br>2013 – 14<br>2013 – 14<br>2013 – 14	74 – 72%<br>82 – 72.6%<br>90% (<1-year)<br>76% (≥1-year)<br>85% (<2 years)	54% VE with 2Ds vs. 48% VE with 1D against rotavirus diarrhoea requiring hospitalisation in children ≥4 months old and 53% VE for at least 1D of the vaccine [92,93]<br>Furthermore, about 23%/22% reduction in the annual gastroenteritis-related hospitalisation/ deaths and 33%/39% reduction in the rotavirus season gastroenteritis-related hospitalisation/deaths were observed in the post-vaccinated era as compared to 9%/0% reduction in non-rotavirus gastroenteritis-related hospitalisation/deaths in 0 – 23 months babies [94]
Burkina Faso	2014 – 16	35 – 76%	There was a reduction of AGE hospitalisation in children <5 years old from 2014 – 15 with 40% and by 2016, it was 44% reduction. Similarly, RAGE hospital admission reduced in 2014 – 15 by 44% and by 2016, it was 54%. However, the vaccine appeared more effective in 6 – 11 months infants (58%) than ≥12 months old children (19%). No trace of indirect effect was observed in aged children [95]
Gambia	2013 – 16	90 – 95%	There was supporting evidence of the efficacy of RV5. RVGE hospitalisation prevalence decreased from 22% in 2013 to 11% in 2015 and 18% in 2016. SRVGE hospitalisation or with intravenous fluids prevalence reduced from 33% in 2013 to 8% in 2015 and 15% in 2016 [96]
Ghana	2012 – 14	93 – 100%	Overall hospital admission for RVI fell from 48% in the pre-RV1 vaccination era to 28% in the post-vaccine era among <5 years children [97]. The previous report of Armah and co-workers with RV5 efficacy against the SRVGE showed 55.5% VE [98]
Kenya	2014 – 17	48 – 56%	Overall VE of 64% against all AGE hospitalisation with RV1 FD. No significant difference in the VE between the age groups (<12 and ≥12 months old children). There was sustainable, significant protection in well-nourished, but very diminished effectiveness in malnourished children [99]
Madagascar	2014 – 16	39 – 69%	All-cause of diarrhoea and rotavirus specific hospitalisation declined. By comparing pre-vaccine with two years post-vaccine era, diarrhoea hospitalisation reduced sequentially from 26% to 16% while rotavirus positivity reduced from 56% to 12% [100]
Malawi	2013 – 15	74.6 – 95.1% (<1-year)<br>18.4 – 87.3% (>1-year)	Rotavirus prevalence declined from 32% in the pre-vaccine era to 24% in the post-vaccine era with a significant decline in the infants (54.2%), but older children remained unchanged [101]<br>Overall VE of 58.3% against RVGE hospitalisation was observed [101]
Mozambique	2016 – 17	76 – 89%	Rotavirus prevalence before vaccine introduction was 40.2% in 2014 and 38.3% in 2015 but reduced to 12.2% and 13.5% in 2016 and 2017 respectively after vaccine introduction. There was a reduction in all-cause of acute diarrhoea hospitalisation in children <12 months old [102]
Rwanda	2012 – 14	50 – 98%	RoV introduction in 2012 brought about a reduction of rotavirus-specific hospital admissions with 61% in 2013 and 70% in 2014. Compared to the pre-vaccine era (2001 – 11), hospital admissions for non-bloody diarrhoea reduced from 17 – 29%, hospital admission for AGE reduced from 48 – 49% and the hospital admission for rotavirus-specific gastroenteritis reduced from 61 – 70%. Reduction in the transmission of RVI among the aged children also demonstrated the herd protection (indirect protection) offered by the RV5 vaccine [103]
Senegal	2014 – 16	89 – 83%	During the sentinel surveillance, 30% of 673 children aged <5 years hospitalised for AGE were rotavirus positive. The 42% proportion of rotavirus-positive hospitalisation in the pre-vaccine era declined by 76 – 10% from 2015 – 16 and 59 – 17% from 2016 – 17. Again, 11% of all hospitalisation cases due to AGE in the pre-vaccine era equally declined by 16 – 9% and 39 – 7% respectively. Significant efficacy against the rotavirus-associated and all-cause of AGE hospitalisations was observed in the infants. Significant reduction from 55 – 70% as observed in the aged children (24 – 59 months old) was evidence of herd immunity [104]
South Africa	2010 – 12	66.5 – 100%	About 60 – 64% reduction in RVI, with 76.9% VE against severe RVGE [105]

Country	*Coverage/ Surveillance*	*Vaccine Coverage*	*Associated vaccine impacts*
Swaziland	2015 – 16	≥36%	Rotavirus positivity reduced from the average of 50.8% in the pre-vaccine era (2013 – 14) to 29% two years post-vaccination with an age-shift from the 10 months old infants in the pre-vaccine era to 13.7 months toddlers in 2016. The seasonal peak for all-cause of diarrhoea and rotavirus-specific hospitalisations in children <5 years old became blunt by 2016. The efficacy of the RV1 in reducing rotavirus positivity among infants <1-year-old was from 49% in 2013 – 14 to 33% in 2016 [106]
Tanzania	2013 – 15	18.7 – 83.8% (<1-year)<br>96.95% (1-year)<br>10.28% (2 – 4 years)	Compared to the pre-vaccine era, there were reductions of 40%, 46%, and 69% in the number of RVI leading to hospitalisation in infants <12 months old and 36%, 26%, and 64% in children <5 years old in 2013, 2014, and 2015 respectively with overall VE of 57% against RVGE hospitalisation [107]
Togo	2014 – 16	75 – 85% (1D)<br>71 – 85% (2Ds)<br>86 – 92%	By 2015, the rate of infants tested positive to rotavirus reduced by 23% and subsequently, 26% in 2016. All-cause AGE hospitalisation declined by 48% among infants <1-year-old and 19% among children 1 to 4 years old by the end of the second year post-vaccine implementation during when the higher level of vaccine coverage was recorded [108]
Zambia	2012 – 13<br>2013 – 14	70 – 58%<br>39 – 77%	Overall VE of 29% for 1D with 70% vaccine coverage and 26% for 2Ds with 58% vaccine coverage against all SRVGE [109]<br>Greater efficacy was observed in infants <1-year-old, which accounted for 84.4% of RVGE hospitalisation cases in the pre-vaccine era. The sequential reduction was from 40.9% in the pre-vaccine era to 34% in 2013 and 26.2% in 2014. Between 2013 – 14, all-cause of diarrhoea and death associated with diarrhoea reduced by 18 – 29% and 27 – 33% respectively [110]
Zimbabwe	2014 – 16	96 – 94%	Rotavirus-positivity decreased from 46% in the pre-vaccine period to 25% in the post-vaccine period. Pre-vaccination (2008 – 13) vs. post-vaccination (2014 – 16) genotype distributions were highly diverse in all the categories of children <5 years old: G9P[8] (34% vs. 11%), G2P[4] (12% vs. 15%), G1P[8] (13% vs. 30%), G9P[6] (3% vs. 20%) and for P-types P[4] (20% vs. 19%), P[6] (25% vs. 35%), P[8] (39% vs. 39%) but there was no difference in the circulating genotypes based on the age groups. Post-vaccination period of RV1 (2015 – 16) witnessed an increasingly prevalent of genotypes G1 and G9 while G2, G8, and G9 were reduced with fairly stable P-type across all the age groups. By 2016, G2P[4] (23%) and G9P[6] (25%) were more prevalent than G1P[8] (14%), and this might be due to vaccine impact.<br>Variations in rotavirus genotypes, including unusual/atypical circulating strains, as compared to the pre-vaccination were mostly attributed to seasonal trends or natural variation. However, there was no convincing evidence of emergent strains among the circulating strains after the introduction of the vaccine. Though few reports showed a reduced efficacy of RV1 against the heterotypic strain G2, however, the efficacy of this vaccine in reducing G2 in Zimbabwe was encouraging and reassuring [111]<br>Mujuru and co-workers concluded that protection beyond one year might be waning and this may not corroborate with their previous finding of about 21 – 33% and 5 – 22% reductions in rotavirus hospitalisations among children 12 – 23 and 24 – 59 months old respectively between 2015 – 16 [112]. Significant reduction in VE was also observed in malnourished children >1-year-old [113]

D/AD/FD = dose/any dose/full doses; **NC** = neighbourhood controls; **NDC** = non-diarrhoea control; **HC** = hospital controls; **TNC** = test-negative control; **GE** = gastroenteritis; **VE** = vaccine effectiveness; **ARD** = acute rotavirus diarrhoea; **AGE** = acute gastroenteritis; **V/SRVGE** = very/severe rotavirus gastroenteritis; **V/SRAGE** = very/severe rotavirus acute gastroenteritis; **RVV** = rotavirus vaccine; **RoVE** = rotavirus vaccine effectiveness; **RVI** = rotavirus infection; **RV1** = Rotarix®; **RV5** = RotaTeq®. Irrespective of the choice of the vaccine, the efficacy produced are very similar in a broadly similar setting.

[a]Panama is among the countries with extremely low vaccine efficacy during their sentinel study [73]. The VE for the Rotarix® ranges from 57 – 85% while for RotaTeq® ranges from 45 – 90% [114]. Even Vietnam and India that used national vaccine produced a combined efficacy of 64% and 55% respectively. RotaSIIL® efficacy was recently accessed in a-3-dose Phase III trial in Niger. By using per-protocol vs. intention-to-treat analysis, the observed results showed 66.7% vs. 69.1%VE against SRVGE; risk of adverse events observed was 68.7% in the vaccine group vs. 67.2% in the placebo, and the risk of serious adverse events observed was 8.3% in the vaccine group vs. 9.1% in the placebo group [115]. Though, no cases of IS reported, however, 27 vs. 22 infants' deaths were recorded in the vaccine vs. placebo groups respectively [115]. The deaths were allegedly declared unconnected to the vaccine trial but may probably be associated with serendipitous infections, metabolic, and nutritional disorders [115].

Table S3. Comparing vaccine effectiveness/efficacy in children by age (<12 months vs. ≥12 months) who mostly received full vaccine doses.

Country	*Vaccine*	*Vaccine effectiveness/efficacy with age (<12 months vs. ≥12 months)*
**Europe**		
Belgium	RV1	91% vs. 90% (against RAGE hospitalisation) [49]
[a]Finland	RV5	92.7% vs. 95.3% (against RVGE EV) [116]
		95.7% vs. 94.0% (against RVGE hospitalisation) [116]
		94.0% vs. 94.7% (against RVGE EV + hospitalisation) [116]
Germany	RV1/RV5	[b]74% vs. 57% (against RVI requiring medical attention) [59]
		[b]80% vs. 85% (against RVGE hospitalisation) [59]
[c]Moldova	RV1	84% vs. 46% (against all RAGE hospitalisation) [60]
		92% vs. 67% (against moderate to severe RAGE hospitalisation) [60]
Spain	RV1/RV5	78% vs. 82% (against RAGE) [64]
		[d]82% vs. 89% (against RAGE hospitalisation) [64]
**North America**		
USA	RV1	85% vs. 91% (against RAGE hospitalisation) [68]
		56% vs. 86% (against RAGE hospitalisation + EV) [69]
		[e]82% vs. 86% (against RAGE hospitalisation) [70]
	RV5	94% vs. 90% (against RAGE hospitalisation) [68]
		[f]85% vs. 89% (against RAGE hospitalisation + EV) [69]
		[e]91% vs. 82% (against severe RAGE) [70]
		93% vs. 78% (against RAGE) [117]
		93% vs. 89% (against RAGE hospitalisation) [71]
		86% vs. 90% (against RAGE hospitalisation) [72]
		63% vs. 81% (1D against RAGE hospitalisation) [72]
		97% vs. 73% (2Ds against RAGE hospitalisation) [72]
**Central America**		
El Salvador	RV1	83% vs. 59% (against RAGE hospitalisation) [74]
		79% vs. 58% (against severe RAGE) [74]
		92% vs. 58% (against very severe RAGE) [74]
Guatemala	RV1/RV5	74% vs. 71% in HC or 73% vs. 53% in TNC (2 – 3Ds vaccines against rotavirus-related diarrhoea hospitalisation or emergency visit) [75]
[c]Nicaragua	RV5	78% vs. 55% (against severe RAGE) [118]
		[g]73% vs. 68% or 64% vs. 30% (against RAGE hospitalisation) [80]
		[g]83% vs. 70% or 70% vs. 33% (against severe RAGE) [80]
		[g]84% vs. 75% or 60% vs. 57% (against very severe RAGE) [80]
**South America**		
Bolivia	RV1	76% vs. 45% (against all RAGE) [81]
		75% vs. 53% (against severe RAGE) [81]
		80% vs. 74% (against very severe RAGE) [81]
		78% vs. 76% (against severe RAGE) [82]
		45% vs. -2% (1D against all severity of AGE) [81]
		45% vs. -5% (1D against SRAGE) [81]

Country	*Vaccine*	*Vaccine effectiveness/efficacy with age (<12 months vs. ≥12 months)*
		40% vs. 56% (1D against VSRAGE) [81]
		[g]77% vs. 76% or 64% vs. 72% (against RAGE hospitalisation) [82]
		[g]78% vs. 76% or 66% vs. 72% (against severe RAGE) [82]
[c]Brazil	RV1	73% vs. -51% (against severe AGE) [119]
		81% vs. 5% (against RAGE hospitalisation) [119]
		[h]95.7% vs. 65.1% or 55.6% vs. 32.1% (against SRVGE) [83]
		[h]88.9% vs. 48% or 60.5% vs. 34.9% (≥1D against SRVGE) [83]
[c]Colombia	RV1	79.19% vs. -39.85% (against all severity of RAGE) [86]
		84.42% vs. -79.49% vs. -2.19%(AAG) (against RAGE hospitalisation) [86]
		62.62% vs. -64.52% vs. -54.18%(AAG) (against severe RAGE) [86]
		66.63% vs. -155.87% vs. -114.28(AAG) (against very severe RAGE) [86]
**Asia**		
Armenia	RV1	68% vs. 60% vs. 62% (both age groups) (against all RVD) [88]
		83% vs. 84% vs. 79% (both age groups) (against severe RVD) [88]
[i]Bangladesh	RV1	45.2% vs. 28.9% (against any severe ARD) [89]
		48.0% vs. 25.8% (against severe ARD caused by homotypic vaccine strains) [89]
**Middle East**		
Israel	RV5	64% vs. 71% (against RAGE hospitalisation) [120]
		49% vs. 76% (1D against RVGE hospitalisation) [120]
		63% vs. 94% (2Ds against RVGE hospitalisation) [120]
		62% vs. 74% (≥1D against RVGE hospitalisation) [120]
		64% vs. 74% (≥2Ds against RVGE hospitalisation) [120]
**Africa**		
Burkina Faso	RV5	59% vs. -7% vs. 36% (AAG) (1D against RVGE hospitalisation) [95]
		-5% vs. -6% vs. -16 (AAG) (2Ds against RVGE hospitalisation) [95]
		58% vs. 19% vs. 35% (AAG) (3Ds against RVGE hospitalisation) [95]
		50% vs. 13% vs. 27% (AAG) (AD against RVGE hospitalisation) [95]
Botswana	RV1	52% vs. 67% (against RAGE hospitalisation) [92]
[c]Ghana	RV1	78% vs. 50% (AD against all RAGE hospitalisation) [97]
		83% vs. 40% (AD against moderate to severe RAGE) [97]
		95% (AD against severe RAGE in <12 months babies)
Kenya	RV1	67% vs. 72% (against RAGE hospitalisation) [99]
[c]Malawi	RV1	70.6% vs. 31.7% (against all RAGE) [101]
Rwanda	RV5	65% vs. 81% (against RAGE hospitalisation) [121]
Rwanda	RV5	65% vs. 81% (against RAGE hospitalisation) [121]
South Africa	RV1	54% vs. 61% (against all RAGE hospitalisation) [105]
Tanzania	RV1	56% vs. 57% (against RAGE hospitalisation) [107]
Zimbabwe	RV1	61% vs. -48% (against any severe RVD hospitalisation/treatment in A&EV) [113]
		60% vs. -47% (AD against any severe RVD hospitalisation/treatment in A&EV) [113]
		68% vs. -38% (against severe RVD hospitalisation/treatment in A&EV) [113]
		68% vs. -37% (AD against severe RVD hospitalisation/treatment in A&EV) [113]

D = dose; **AD** = any dose; **FD** = full doses; **A&EV** = accident & emergency visit; **NDC** = non-diarrhoea controls; **NC** = neighbourhood controls; **HC** = hospital controls; **EV** = emergency visit; **TNC** = test-negative controls; **AAG** = all age group; **AGE** = acute gastroenteritis; **RVD** = rotavirus diarrhoea; **ARD** = acute rotavirus diarrhoea; **V/SRAGE** = very/severe rotavirus acute gastroenteritis; **V/S/RVGE** = very severe rotavirus gastroenteritis; **ITT** = intent-to-to treat population; **ATP** = according to protocol.

[a]Vaccine efficacy was measured further in 24 to 35 months as 100% against RVGE EV, 80.2% against RVGE hospitalisations, and 85.9% against RVGE EV + hospitalisation. The overall efficacy was 94% each against RVGE EV, hospitalisation and RVGE EV + hospitalisations. This assessment was conducted for 3.1 years [116].

[b]The report was for aged 6 – 17 months vs. 18 – 29 months. However, the overall vaccine efficacy across all the children age group (6 to 29 months old) was 68% against RVI that required medical attention and 80% against hospitalisation [59].

[c]VE appeared significantly higher in children in <12 months age bracket than ≥12 months old in Brazil, Colombia, El Salvador, and Moldova, which are rated moderate mortality rate. Moldova report claimed no significant difference, and the extremely low VE in Colombia was attributed to low sample size and high vaccine coverage vis-à-vis diarrhoea caused by RV1 vaccination in the case-control. Ghana, Malawi, and Nicaragua also have VE significantly higher in children <12 months than ≥12 months which are rated high mortality rate.

Few of the MICs and LICs showed a positive correlation between the number of doses and VE in these two comparable age groups, for example, RV5 in Israel. However, a report from Bolivia showed a drastic reduction in VE in the assessment of 1D of RV1 in children <12 months old compared to children ≥12 months, though there was evidence of increased VE with clinical severity in children ≥12 months old [81]. Report from Botswana showed no significant difference in VE irrespective of the number of vaccine doses and ages [92].

[d]The report was for <24 vs. ≥24 months [64].

[e]Reports for VE in 3 to 7 years old children were also observed. The VE gradually decreased in the ascending order of age but not statistically different from VE observed in one and two years old children: 91%, 82%, 88%, 76%, 60%, and 69% for 6 to 7 years old children immunised with RV5. This trend was similarly observed with RV1 for 1 to 4 years old children: 82%, 86%, 80%, and 58% respectively in the increasing order of age [70]. Cortese and co-workers reported VE of 91% against RAGE hospitalisation in babies ≥24 months in the USA [71].

[f]Further reports made available for three and four years old children were not statistically different from one and two years old for RV5. The VE in ascending order of age against rotavirus hospitalisations and EV was 85%, 89%, 83%, and 79% respectively. There was no evidence of waning over four years or two years of follow-up for RV5 and RV1, respectively [69].

[g]Reports were compared against NDC or TNC, respectively in Nicaragua and Bolivia. In Nicaragua, VE in all ages (≥6 years old children) under rotavirus hospitalisation and all Vesikari scales was not statistically different from each sub-age groups with either NDC or TNC: 70% vs. 45% against rotavirus hospitalisation, 76% vs. 51% against severe diarrhoea (Vesikari ≥11), and 79% vs. 58% against very severe diarrhoea (Vesikari ≥15).

[h]Reports were based against the NC or HC.

[i]This was the total VE estimated from modified ATP analysis. The total VE estimated from modified ITT against any severe RVD in all age bracket (6 to 20 weeks old) was 38.7% or 41.4% as estimated from modified ATP. The total effectiveness of RV1 against homotypic and heterotypic strains in all age bracket was 48.1% vs. 43.0%. The VE against all severe cases in all age bracket was 42.8%. The overall VE against ARD of any severity and severe ARD among eligible children <2 years old was between 20 – 29%.

Table S4. Comparing vaccine effectiveness/efficacy in sequential vaccine doses against rotavirus infection.

Country	*Vaccine*	*Vaccine effectiveness/efficacy (FD vs. 1D vs. 2Ds)*
**Europe**		
Belgium	RV1	90% vs. 91% (against RAGE hospitalisation) [49]
Finland	RV5	[a]98.7% vs. 98.8% (against EV in the 1st year) [116]<br>[a]96.1% vs. 94.9% (against RVGE hospitalisation in 1st year) [116]<br>[a]94.7% vs. 94.0% (against EV & RVGE hospitalisation in the 1st year) [116]<br>[a]96.0% vs. 97.7% (against EV in the 2nd year) [116]<br>[a]86.9% vs. 90.8% (against RVGE hospitalisation in the 2nd year) [116]<br>[a]93.8% vs. 93.9% (against EV & RVGE hospitalisation in the 2nd year) [116]<br>[a]93.9% vs. 94.0% (against over EV) [116]<br>[a]95.6% vs. 94.0% (against overall RVGE hospitalisation) [116]<br>[a]94.6% vs. 94.0% (against overall EV & RVGE hospitalisation) [116]<br>[b]100% (against EV in the 3rd year) [116]<br>[b]100% (against EV & RVGE hospitalisation in the 3rd year) [116]
Moldova	RV1	79% vs. 60% vs. (75% overall) (against all RAGE hospitalisation) [60]<br>84% vs. 71% vs. (82% overall) (against moderate to severe RAGE hospitalisation) [60]
Portugal	RV1	81.3% vs. (81.3% for ≥1D) (against RVGE hospital attendance) [62]<br>94.9% vs. (96.1% for ≥1D) (against RVGE hospital admission) [62]
	RV5	85.3% vs. (86.6% for ≥1D) (against RVGE hospital attendance) [62]<br>[c]100% vs. (96% for ≥1D) (against severe RVGE hospital admission) [62]
[d]Spain	RV1	75% vs. 76% (against RAGE) [64]<br>97.2% vs. 96% (against any RVGE) [63]<br>97.3% vs. 97.5% (against RVGE hospitalisation) [63]<br>43.8% (against any RVGE with partially vaccination) [63]<br>70.7% (against RVGE hospitalisation with partial vaccination) [63]<br>84% vs. 89% (against RAGE hospitalisation) [122]
	RV5	81% vs. (80% for AD) (against RAGE) [64]<br>84.4% vs. (85.1% for ≥1D) (against any RVGE) [63]<br>95% vs. (92.9% for ≥1D) (against RVGE hospitalisation) [63]<br>87.5% (against any RVGE with partial vaccination) [63]<br>87% (against RVGE hospitalisation with partial vaccination) [63]<br>91% vs. 84% vs. 89% (against RAGE hospitalisation) [122]
**North America**		
Canada	RV1	91.2% vs. (92.5% for ≥1D) (against RAGE) [67]
USA	RV1	70% vs. 57% (against RAGE hospitalisation) [69]<br>80% vs. 96% (against RAGE hospitalisation) [70]<br>91% (against RAGE hospitalisation) [68]

Country	*Vaccine*	*Vaccine effectiveness/efficacy (FD vs. 1D vs. 2Ds)*
	RV5	84% vs. 70% vs. 78% (against RAGE hospitalisation) [69]
		80% vs. 68% vs. 78% (against RAGE hospitalisation) [70]
		92% (against RAGE hospitalisation) [68]
		[e]95% (against RAGE hospitalisation) [68]
		95% vs. 89% vs. 89% (against RAGE hospitalisation) [72]
		74% vs. 75% vs. 82% (against RAGE emergency visit) [72]
		89% vs. 65% vs. 82% (against RAGE hospitalisation) [123]
		92% vs. 68% vs. 83% (against RAGE) [124]
**Central America**		
El Salvador	RV1	76% vs. 51% (against RAGE hospitalisation) [74]
		[f]73% vs. 36% (against severe RAGE) [74]
		[f]83% vs. 45% (against very severe RAGE) [74]
Guatemala	RV1/RV5	[g]63% (with RV1) vs. 69% (with RV5) (against RVGE hospitalisation or EV) [75]
		[h]51% (with RV1) vs. 43% (with RV5) (against RVGE hospitalisation or EV) [75]
		[i]59% vs. 60% (1D of either RV1/RV5 against RVGE hospitalisation or EV) [75]
		[i]64% vs. 55% (≥1D of either RV1/RV5 in ITV against RVGE hospitalisation or EV) [75]
		[i]74% vs. 52% (2 – 3Ds of RV1/RV5 against RVGE hospitalisation or EV) [75]
		[i]70% vs. 54% (≥1D against RVGE hospitalisation or EV) [75]
	RV5	69% vs. (74% for 2/3Ds) vs. (70% for ≥1D) in HC or 43% vs. (52% for 2/3Ds) vs. (54% for ≥1D) in TNC (against hospitalisation or EV) [75]
Mexico	RV1	94% vs. 84% (against RAGE hospitalisation) [125]
Nicaragua	RV5	[j]46% vs. 52% vs. 51% (against all RAGE hospitalisation or with IVH) [78]
		[k]13% (against RAGE hospitalisation) [78]
		[k]58% (against severe RAGE) [78]
		[k]77% (against very severe RAGE) [78]
**South America**		
Bolivia	RV1	[l]77% vs. 56% or 69% vs. 36% (against RAGE hospitalisation) [82]
		[l]76% vs. 54% or 69% vs. 34% (against severe RAGE) [82]
		[l]74% vs. 40% or 62% vs. 6% (against very severe RAGE) [82]
		[f]59% vs. 26% (against all severity of RAGE) [81]
		[f]54% vs. 16% (against severe RAGE) [81]
		[f]72% vs. 8% (against very severe RAGE) [81]
Brazil	RV1	76% vs. 62% (against RAGE hospitalisation) [126]
		[m]72.7% and 25.6% (against mild to moderate RVGE) [83]
		[m]78.8% and 53.7% (against severe RVGE) [83]
		[m]90% and 28.6% (against very severe RVGE) [83]
**Asia**		
[n]India	116E	34.6% (1st year) vs. 36.4% (2nd year) (against any severity of RVGE)[127,128]
		24.1% (1st year) vs. NA (2nd year) (against SGE of any cause) [127,128]
		56.4% (1st year) vs. 55.1% (2nd year) (against SRVGE) [127,128]
		49.8% (1st year) vs. 57.2% (2nd year) (against VSRVGE) [127,128]
		56.4% (1st year) vs. 55.6% (2nd year) (against SRVGE hospitalisation/rehydration therapy [127,128]

Country	*Vaccine*	*Vaccine effectiveness/efficacy (FD vs. 1D vs. 2Ds)*
		32.9% (1st year) vs. NA (2nd year) (against RVGE of any severity with hospitalisation/rehydration therapy [127,128]
oIndia	BRV-PV	5/4.6% vs. 7.5/5.1% (against SGE of any aetiology) [129]<br>28.3/22.6% vs. 35.5/24.2% (against RVGE of any severity) [129]<br>36/39.5% vs. 41.9/38.8% (against SRVGE) [129]<br>34.1/32.9% vs. 40.6/36.6% (against SRVGE in the 1st year) [129]<br>34.6/38.9% vs. 40.8/38.2% (against SRVGE vaccine serotypes) [129]<br>34.8/33.4% vs. 42.2/35.1% (against SRVGE hospitalisation) [129]<br>60.5/54.7% vs. 61.3/52.9% (against VSRVGE) [129]
**Middle East**		
Israel	RV1	50% vs. 53.9% vs. (50.1% for AD) (against RAGE) [90]<br>p33.8% vs. 31.8% vs. (33.6% for AD) (against RAGE in the low-medium socioeconomic status) [90]<br>p55.4% vs. 60.3% vs. (55.8% for AD) (against RAGE in the medium-high socioeconomic status) [90]
	RV5	63% vs. 56% vs. 73% vs. (64% for ≥1D) vs. (65% for ≥2Ds) (against RAGE hospitalisation in all children) [120]<br>62% vs. 41% vs. 70% vs. (61% for ≥1D) vs. (64% for ≥2Ds) (against RAGE hospitalisation in Bedouin children only) [120]
**Africa**		
Botswana	RV1	53% vs. 54% (against severe RAGE) [92]<br>70% vs. 90% (against very severe RAGE) [92]
qBurkina Faso	RV5	39% vs. 39% vs. 13% vs. (35% for AD) (against SRVGE) [95]<br>38% vs. 38% vs. -13% vs. (37% for AD) (against VSRVGE) [95]<br>35% vs. 36% vs. -16% vs. (27% for AD) (against rotavirus hospitalisations in infants ≥6 months old) [95]<br>58% vs. 59% vs. -5% vs. (50% for AD) (against rotavirus hospitalisations in infants ≥6 and <12 months old) [95]<br>19% vs. -7% vs. -6% vs. (13% for AD) (against rotavirus hospitalisations in infants ≥12 months old) [95]
rGhana	RVI	18% vs. (60% for AD) (against all RAGE hospitalisation) [97]<br>26% vs. (62% for AD) (against moderate to severe RAGE hospitalisation) [97]<br>52% vs. (80% for AD) (against severe RAGE hospitalisation) [97]
Kenya	RV1	64% vs. 54% vs. (58% for ≥1D) (against RAGE hospitalisation) [99]<br>67% (against less severe RAGE) [99]<br>61% (against severe RAGE) [99]
Malawi	RV1	64% vs. 65% (against RAGE) [130]<br>68% vs. 69% (against severe RAGE) [130]<br>66.3% (against moderate to severe RAGE hospitalisation) [101]<br>59.7% (against severe RAGE) [101]<br>65.2% (against very severe RAGE) [101]
Niger	BRV-PV	66.7% vs. (69.1% for ≥1D) (against SRVGE for the RotaSIIL®) [115]
kRwanda	RV5	75% (against all RAGE) [121]<br>80% (against RAGE hospitalisation) [121]
South Africa	RV1	57% vs. 40% (against all RAGE hospitalisation) [105]
kTanzania	RV1	61% (against moderate RVGE) [107]<br>62% (against severe RVGE) [107]<br>69% (against very severe RVGE) [107]

Country	*Vaccine*	*Vaccine effectiveness/efficacy (FD vs. 1D vs. 2Ds)*
Zambia	RV1	[s]26% vs. 29% (or 27% for ≥1D against all severe RAGE) [109]<br>56% vs. 62% (or 57% for ≥1D against RAGE hospitalisation) [109]<br>5% vs. 77% (or 17% for ≥1D against severe RAGE) [109]<br>[s]48% vs. ND (or 60% for ≥1D against very severe RAGE) [109]

AD = any dose; **FD** = full doses; **NA** = not available; **ND** = not determined; **ITTP** = intention-to-treat population; **PPP** = per-protocol population; **ITV** = intention-to-vaccinate; **NDC** = non-diarrhoea control; **NC** = neighbourhood controls; **HC** = hospital controls; **TNC** = test-negative controls; **EV** = emergency visit; **REST** = rotavirus efficacy and safety trial; **FES** = Finnish extension study; **SGE** = severe gastroenteritis; **SRVGE** = severe rotavirus gastroenteritis; **VSRVGE** = very severe rotavirus gastroenteritis; **RVGE/RAGE** = rotavirus gastroenteritis/rotavirus acute gastroenteritis; **IVH** = intravenous hydration

A standalone VE or efficacy means FD vaccine only

[a]Analysis was done by comparing VE of the REST vs. REST+FES

[b]VE analysis was done with REST+FES only

[c]Portugal recorded no case of admission after a FD of RV5

[d]In Spain, AD vaccination (RV1/RV5) vs. FD (RV1/RV5) produced VE of 78% each. Also, in the diagnostic period, FD produced 64% VE in 2008 – 09 while in 2010 – 11, the VE was 83%. Against hospitalisation, overall FD (RV1/RV5) produced VE of 83%, 39% (between 2008 – 09), and 96% (between 2010 – 11)

[e]Reports for 2D-mixed vaccination (1 dose RV1 + 1 dose RV5)

[f]Effect of incomplete dosages was highly noticeable

[g]Analysis of the VE by comparing to the HC (with RV1) vs. HC (with RV5)

[h]Analysis of the VE by comparing to the TNC (with RV1) vs. TNC (with RV5)

[i]Analysis of the VE by comparing to the HC vs. TNC (with either RV1 or RV5)

[j]Neither the hospitalisation cases only (44% vs. 55% vs. 44%) nor IVH cases only (48% vs. 54% vs. 52%) was statistically different from the combined cases stated in the table

[k]A typical case of increased VE with clinical severity after a FD vaccine

[l]The authors claimed no statistical difference, and the cases were compared with either NDC or TNC. However, the VE appeared lower when 1D of RV1 in the TNC was compared to the 2Ds vaccine

[m]This analysis involved only the FD and the VE was estimated with reference to the NC and HC respectively

[n]The difference between the 1st and 2nd year efficacy of Indian 116E (Rotavac®) as well as the overall efficacy was not statistically different from either the 1st or the 2nd year vaccine efficacy after the FD vaccine regardless of the analysis (ITT or PPP analysis)

[o]The efficacy at 1st/2nd year of life after 3Ds of Indian BRV-PV (RotaSIIL®) vaccine was compared under PPP vs. ITTP analysis

[p]Evidence of higher VE in the medium-high socioeconomic status as compared to the low-medium socioeconomic status

[q]In Burkina Faso, low VE was observed. Notwithstanding, moderate effectiveness was observed FD only in infants <12 months old. There was a relatively lower VE against RVI with Vesikari ≥11 and ≥15

[r]Here, one or more doses of RV1 appeared to produce more significant effects than full doses

[s]One of the evidence of lower VE in Africa, and there was no correlation between the VE and severity of RVI.

Table S5. Attributed/relative risk associated with rotavirus vaccine doses.

Vaccines	Cases represent the associated risk of intussusception/age range in weeks/days risk window in vaccinated children (analysis performed)				References
	1D	2Ds	3Ds	Any dose or *all doses*	
RRV-TV	†1.0/6 – 30/NA (direct comparison)<br>†4.6/6 – 30/NA (trend comparison)				USA [131]
RRV-TV*			5.7/4 – 44/3 – 7 (incidence rates)<br>1.1/4 – 44/8 – 14 (incidence rates)<br>22.70/4 – 44/3 – 7 (relative risk)<br>4.40/4 – 44/8 – 14 (relative risk)		USA [132]
RRV-TV*	58.9/4 – <52/3 – 7 (case-series analysis)<br>37.2/4 – <52/3 – 7 (case-control analysis)<br>29.4.9/4 – <52/3 – 14 (case-series analysis)<br>21.7/4 – <52/3 – 14 (case-control analysis)<br>9.4/4 – <52/8 – 14 (case-series analysis)<br>8.2/4 – <52/8 – 14 (case-control analysis)<br>2.3/4 – <52/15 – 21 (case-series analysis)<br>1.1/4 – <52/15 – 21 (case-control analysis)<br>†29.4/4 – <52/3 – 14 (case-series analysis)<br>†21.7/4 – <52/3 – 14 (case-control analysis)	11/4 – <52/3 – 7 (case-series analysis)<br>3.8/4 – <52/3 – 7 (case-control analysis)<br>6.8/4 – <52/3 – 14 (case-series analysis)<br>3.3/4 – <52/3 – 14 (case-control analysis)<br>3.8/4 – <52/8 – 14 (case-series analysis)<br>1.8/4 – <52/8 – 14 (case-control analysis)<br>1.4/4 – <52/15 – 21 (case-series analysis)<br>0.9/4 – <52/15 – 21 (case-control analysis)		14.4/4 – <52/3 – 7 (case-control)<br>10.6/4 – <52/3 – 14 (case-control)<br>5.3/4 – <52/8 – 14 (case-control)<br>1.1/4 – <52/15 – 21 (case-control)	USA [133]
RV1*	†2.4/5 – 36.9/1 – 7<br>†2.4/5 – 36.9/1 – 21<br>†1.6/5 – 36.9/1 – 21 (cohort comparison)	†4.3/5 – 36.9/1 – 7<br>†3.7/5 – 36.9/1 – 21<br>†7.3/5 – 36.9/1 – 21 (cohort comparison)		*†3.1/5 – 36.9/1 – 7*<br>*†2.8/5 – 36.9/1 – 21*<br>*†3.7/5 – 36.9/1 – 21 (cohort comparison)*	USA [134]
RV5	†1.1/5 – 36.9/1 – 7<br>†1.5/5 – 36.9/1 – 21<br>†1.2/5 – 36.9/1 – 21 (cohort comparison)	†0.4/5 – 36.9/1 – 7<br>†-0.1/5 – 36.9/1 – 21<br>†-0.2/5 – 36.9/1 – 21 (cohort comparison)	†0.6/5 – 36.9/1 – 7<br>†-0.05/5 – 36.9/1 – 21<br>†-0.3/5 – 36.9/1 – 21 (cohort comparison)	*†0.8/5 – 36.9/1 – 7*<br>*†0.6/5 – 36.9/1 – 21*<br>*†0.4/5 – 36.9/1 – 21 (cohort comparison)*	
RV1*	8.82/4 – 34/1 – 7 (historical comparison)	8.17/4 – 34/1 – 7 (historical comparison)<br>9.4/4 – 34/ 1 – 7 (r.r compared with 2Ds RV5)<br>5.3†/4 – 34/1 – 7		*8.38/4 – 34/1 – 7 (historical comparison)*	USA [135]
RV5	2.63/4 – 34/1 – 7 (historical comparison)	0/4 – 34/1 – 7 (historical comparison)	1.25/4 – 34/1 – 7 (historical comparison)	*1.13/4 – 34/1 – 7 (historical comparison)*	
RV1 or RV5	3.07/4 – 34/1 – 7 (relative risk analysis)	NA	NA	*9.1/4 – 34/1 – 7 (relative risk)*	
RV1*	3.45/4 – <36/1 – 7 (relative risk analysis)<br>1.53/4 – <36/1 – 21 (relative risk analysis)	1.05/4 – <36/1 – 7 (relative risk analysis)<br>0.88/NA/4 – <36 (relative risk analysis)		1.58/4 – <36/1 – 7 (relative risk)<br>1.37/NA/4 – <36 (relative risk)	Australia [136]
RV5*	5.26/4 – <36/1 – 7 (relative risk analysis)<br>3.51/4 – <36/1 – 21 (relative risk analysis)	1.33/4 – <36/1 – 7 (relative risk analysis)<br>0.67/4 – <36/1 – 21 (relative risk analysis)	0/4 – <36/1 – 7 (relative risk analysis)<br>0/4 – <36/1 – 21 (relative risk analysis)	1.15/4 – <36/1 – 7 (relative risk)<br>0.77/4 – <36/1 – 21 (relative risk)	
(RV1+RV5)*‡				‡1.7 vs. 0.8 vs. 1.3 vs. 1.5 (1 – <12)<br>‡3.9 vs. 3.8 vs. 3.9 vs. 4.1 (1 – <12)<br>‡8.6 vs. 5.3 vs. 7.2 vs. 7.8 (12 – <20)<br>‡7.5 vs. 9.9 vs. 8.5 vs. 10.3 (12 – <20)<br>‡10.3 vs. 12.2 vs. 11.1 vs. 12.3 (20 – <28)<br>‡12 vs. 12.7 vs. 12.3 vs. 12.5 (20 – <28)<br>‡6.9 vs. 15.7 vs. 10.7 vs. 10.5 (28 – <36)<br>‡12 vs. 14.2 vs. 12.9 vs. 12.3 (28 - <36)	
RV1*	6.76/4 – <52/1 – 7 (case-series analysis)<br>15.61/4 – <52/1 – 7 (case-control analysis<br>3.45/4 – <52/8 – 21 (case-series analysis)<br>6.48/4 – <52/8 – 21 (case-control analysis)	2.84/4 – <52/1 – 7 (case-series analysis)<br>2.44/4 – <52/1 – 7 (case-control analysis)<br>2.11/4 – <52/8 – 21 (case-series analysis)<br>1.35/4 – <52/8 – 21 (case-control analysis)			Australia [137]

**Vaccines**	**Cases represent the associated risk of intussusception/age range in weeks/days risk window in vaccinated children (analysis performed)**				**References**
	**1D**	**2Ds**	**3Ds**	**Any dose or *all doses***	
RV5*	9.89/4 – <52/1 – 7 (case-series analysis)<br>11.74/4 – <52/1 – 7 (case-control analysis)<br>6.32/4 – <52/8 – 21 (case-series analysis)<br>4.65/4 – <52/8 – 21 (case-control analysis)	2.81/4 – <52/1 – 7 (case-series analysis)<br>2.53/4 – <52/1 – 7 (case-control analysis)<br>1.77/4 – <52/8 – 21 (case-series analysis)<br>1.38/4 – <52/8 – 21 (case-control analysis)	0.75/4 – <52/1 – 7 (case-series)<br>1.06/4 – <52/1 – 7 (case-control)<br>0.56/4 – <52/8 – 21 (case-series)<br>0.8/4 – <52/8 – 21 (case-control)		
RV1*	5.3/6 – 35/1 – 7 (case-series analysis)<br>5.8/6 – 35/1 – 7 (case-control analysis)<br>1.1/6 – 35/8 – 14 (case-series analysis)<br>1/6 – 35/8 – 14 (case-control analysis)<br>0.9/6 – 35/15 – 21 (case-series analysis)<br>0.8/6 – 35/15 – 21 (case-control analysis)	1.8/6 – 35/1 – 7 (case-series analysis)<br>1.1/6 – 35/1 – 7 (case-control analysis)<br>2.2/6 – 35/8 – 14 (case-series analysis)<br>2.3/6 – 35/8 – 14 (case-control analysis)<br>2.2/6 – 35/15 – 21 (case-series analysis)<br>2/6 – 35/15 – 21 (case-control analysis)		1.0/6 – 35/1 – 7 (case-control analysis)	Mexico [138]
	1.1/6 – 35/1 – 7 (case-series analysis)<br>1.4/6 – 35/1 – 7 (case-control analysis)<br>1.3/6 – 35/8 – 14 (case-series analysis)<br>1.6/6 – 35/8 – 14 (case-control analysis)<br>0.2/6 – 35/15 – 21 (case-series analysis)<br>0.6/6 – 35/15 – 21 (case-control analysis)	2.6/6 – 35/1 – 7 (case-series analysis)<br>1.9/6 – 35/1 – 7 (case-control analysis)<br>1.4/6 – 35/8 – 14 (case-series analysis)<br>0.9/6 – 35/8 – 14 (case-control analysis)<br>0.9/6 – 35/15 – 21 (case-series analysis)<br>0.8/6 – 35/15 – 21 (case-control analysis)		1.7/6 – 35/1 – 7 (case-control analysis)	Brazil [138]
RV1*	6.49/<52/0 – 6<br>3.24/<52/0 – 15<br>1.75/<52/0 - 30<br>†3 – 4/<52/1 – 7	1.29/<52/0 – 6<br>1.06/<52/0 – 15<br>1.06/<52/0 - 30			Mexico [139]
RV1*	13.81/6 – 26/1 – 7 (historical comparison)<br>1.59/6 – 26/8 – 21 (historical comparison)<br>4.53/6 – 26/1 – 21 (historical comparison)<br>8.5/6 – 26/1 – 7 (nonhistorical comparison)<br>1.18/6 – 26/8 – 21 (nonhistorical comparison)<br>3.13/6 – 26/1 – 21 (nonhistorical comparison)<br>1.68†/6 – 26/1 – 7<br>1.91†/6 – 26/1 – 21	2.2/6 – 26/1 – 7 (historical comparison)<br>2.77/6 – 26/8 – 21 (historical comparison)<br>2.6/6 – 26/1 – 21 (historical comparison)<br>1.74/6 – 26/1 – 7 (nonhistorical comparison)<br>2.74/6 – 26/8 – 21 (nonhistorical comparison)<br>2.41/6 – 26/1 – 21 (nonhistorical comparison)<br>1.49†/6 – 26/1 – 21			England [140]
RV1*	8.36/<52/1 – 7 (age(d)-adjusted analysis)<br>7.81/<52/1 – 7 (age(m)-adjusted analysis)<br>1.55†/<52/1 – 7	3.09/<52/1 – 7 (age(d)-adjusted)<br>3.02/<52/1 – 7 (age(m)-adjusted)<br>1.54/<52/8 – 21 (age(d)-adjusted)<br>1.51/<52/8 – 21 (age(m)-adjusted)			Singapore [141]
RV5	NA<br>NA<br><br>1.21/4 – 34/1 – 7 (incidence ratio analysis)<br>1.23/4 – 34/1 – 30 (incidence ratio analysis)<br>†1.53/4 – 34/1 – 30 (historical comparison)	0/4 – 34/1 – 7 (relative risk analysis)<br>0.36/4 – 34/1 – 30 (relative risk analysis)<br><br>0.62/4 – 34/1 – 7 (incidence ratio analysis)<br>0.97/4 – 34/1 – 30 (incidence ratio analysis)	1.57/4 – 34/1 – 7 (relative risk)<br>1.57/4 – 34/1 – 30 (relative risk)<br><br>1.05/4 – 34/1 – 7 (incidence ratio)<br>0.88/4 – 34/1 – 30 (incidence ratio)	*0.9/4 – 34/1 – 7 (relative risk analysis)*<br>*0.95/4 – 34/1 – 30 (relative risk analysis)*<br><br>*0.92/4 – 34/1 – 7 (incidence ratio)*<br>*1.01/4 – 34/1 – 30 (incidence ratio)*	USA [142]
RV5	1.0/6 – 12/0 – 30 (compd. to DTaP infant)<br>1.5/6 – 12/0 – 60 (compd. to DTaP infant)<br>0.9/6 – 12/0 – 30 (compd. to hist. DTaP)<br>1.4/6 – 12/0 – 60 (compd. hist. DTaP)	1.4/6 – 12/0 – 30 (compd. to DTaP infant)<br>1.8/6 – 12/0 – 60 (compd. to DTaP infant)<br>0.9/6 – 12/0 – 30 (compd. to hist. DTaP)<br>0.4/6 – 12/0 – 60 (compd. to hist. DTaP)	0/6 – 12/0 – 30 (compd. to DTaP infant)<br>0/6 – 12/0 – 60 (compd. to DTaP infant)<br>NA<br>NA	0.8/6 - 12/0 – 30 (compd. to DTaP infant)<br>1.3/6 - 12/0 – 60 (compd. to DTaP infant)<br>0.9/6 - 12/0 – 30 (compd. to hist. DTaP)<br>0.6/6 - 12/0 – 60 (compd. to hist. DTaP)	USA [143]

compd. = compared; **D = dose; d** = days; **DTaP** = concurrent and historical cohorts receiving *diphtheria-tetanus-acellular pertussis*; **hist.** = historical; **m** = months; **NA** = not available; r.r = relative risk

*significant increase risk of IS was associated with the corresponding vaccine dose; often observed in the first dose

†Attributable risk/100, 000 vaccinated infants

‡Incidence rates of intussusception/10, 000 in 2 states, RV1 (2 states) vs. RV5 (2 states) vs. 4 states combined vs. All Australian states (age range).

Table S6. Summary of vaccine effectiveness against the specific/dominant circulating genotype in some selected countries (vaccinations are mostly full doses unless otherwise stated).

Country	*Vaccine*	*Genotypes*	*Vaccine effectiveness*
**Europe**			
Belgium	RV1	G1P[8]	95% [49]
		G2P[4]	85% [49]
Finland	RV5	G1	[a]94.1% vs. 95.1% (against RVGE EV) [116]
			[a]96.7% vs. 96.0% (against RVGE hospitalisation) [116]
			[a]95.1% vs. 95.5% (against RVGE EV & hospitalisation) [116]
		G2	[a]83.4% vs. 85.8% (against RVGE EV) [116]
			[a]100% vs. 75.2% (against RVGE hospitalisation) [116]
			[a]87.6% vs. 81.9% (against RVGE EV & hospitalisation) [116]
		G3	[a]100% vs. 90.1% (against RVGE EV) [116]
			[a]83.4% vs. 87.6% (against RVGE hospitalisation) [116]
			[a]93.4% vs. 89.0% (against RVGE EV & hospitalisation) [116]
		G4	[a]80.1% vs. 85.8% (against RVGE EV) [116]
			[a]92.3% vs. 82.5% (against RVGE hospitalisation) [116]
			[a]89.1% vs. 83.4% (against RVGE EV & hospitalisation) [116]
		G9	[a]100% vs. 100% (against RVGE EV) [116]
			[a]100% vs. 87.6% (against RVGE hospitalisation) [116]
			[a]100% vs. 94.2% (against RVGE EV & hospitalisation) [116]
		G1 – G4	[a]85.4% vs. 85.7% (against RVGE EV in 1st year) [116]
			[a]92.7% vs. 92.6% (against RVGE hospitalisation in 1st year) [116]
			[a]88.6% vs. 88.8% (against RVGE EV & hospitalisation in 1st year) [116]
			[a]92.8% vs. 94.0% (against RVGE EV in 2nd year) [116]
			[a]89.9% vs. 89.9% (against RVGE hospitalisation in 2nd year) [116]
			[a]92.3% vs. 92.1% (against RVGE EV & hospitalisation in 2nd year) [116]
			[a]86.4% vs. 87.6% (against overall RVGE EV) [116]
			[a]92.6% vs. 92.2% (against overall RVGE hospitalisation) [116]
			[a]88.9% vs. 89.7% (against overall RVGE EV & hospitalisation) [116]
			[b]100% (against RVGE EV in 3rd year) [116]
			[b]100% (against RVGE hospitalisation in 3rd year) [116]
			[b]100% (against RVGE EV & hospitalisation in 3rd year) [116]

Country	*Vaccine*	*Genotypes*	*Vaccine effectiveness*
**North America**			
USA	RV1	G1P[8]	89% [68]
		G2P[4]	94% [68]
		G3P[8]	74% [69]
	RV5	G1P[8]	95% [68], 89% [69,70]
		G2P[4]	98% [68], 87% [69,70]
		G3P[8]	87% [69], 80% [70]
		G12P[8]	83% [69], 78% [70]
	RV5	G1	96% (AD) vs. 95% (1Ds) vs. 93% (2Ds) vs. 99% (3Ds) [72]
		G2	72% (AD) [72]
		G3	86% (AD) vs. 76% (2Ds) vs. 84% (3Ds) [72]
		G9	83% (AD) [72]
		G12	90% (AD) [72]
		G1 – G4	87% (AD) vs. 85% (1D) vs. 79% (2Ds) vs. 91% (3Ds) [72]
		G1 – G4, G9, G12	84% (AD) vs. 74% (1D) vs. 88% (2Ds) vs. 87% (3Ds) [72]
**Central America**			
Guatemala	RV1/RV5	G12P[8]	74% with 2 – 3Ds comparing with HC [75]
			54% with 2 – 3Ds comparing with TNC [75]
Mexico	RV1	G9P[4]	94% [125]
Nicaragua	RV5	G1	63% [118]
		G2	65% [118]
		G3	100% [118]
		G4	88% [118]
		P[8]	65% [118]
		P[4]	64% [118]
		P[5]	100% [118]
		P[6]	84% [118]
**South America**			
Bolivia	RV1	G2P[4]	69% (NDC) or 59% (TNC) [82]
		G3P[8]	93% (NDC) or 74% (TNC) [82], 48% (all Vesikari scores) [81]
		G9P[6]	87% (NDC) or 80% (TNC) [82]
		G9P[8]	85% (NDC) or 80% (TNC) [82]
		G9P[8]	90% (NDC) or 82% (TNC) for 6 – 11 months and 82% (NDC) or 78% (TNC) for ≥12 months children [82]
		G9P[8]	77% (any SRVGE) vs. 80% (VSRVGE) [81]
Brazil	RV1	[c]G2P[4]	77% in 6 – 11 months old babies with severe RAGE [119]
			85% in 6 – 11 months babies with RAGE hospitalisation [119]
			-24% in >12 months old babies with severe RAGE [119]
			5% in >12 months babies with RAGE hospitalisation [119]
			75.4% (NC) and 38.9% (HC) [83]
	RV1	G1P[8]	90% (<12 months age) vs. 89% (1 to 2 years) [126]
		G1P[8]	31% (1D) vs. 89% (FD) [126]

Country	*Vaccine*	*Genotypes*	*Vaccine effectiveness*
		G2P[4]	77% (<12 months age) vs. 75% (1 to 2 years) [126]
		G2P[4]	57% (1D) vs. 76% (FD) [126]
		G1	38% (1D) vs. 74% (FD) [126]
		G2	56% (1D) vs. 76% (FD) [126]
**Asia**			
[d]Bangladesh	RV1	G1P[8]	54.5% [89]
		G2P[4]	45.8% [89]
		G9P[8]	20.0% [89]
		G12P[6]	36.4% [89]
		G12P[8]	51.5% [89]
Bangladesh & Vietnam	RV5	G1	46.2% [144]
		G2	29.2% [144]
		G3	67.0% [144]
		G9	48.7% [144]
		P1A[8]	49.7% [144]
		P1B[4]	40.9% [144]
		P2A[6]	60.5% [144]
India	116E	G1P[8]	[e]31.3% (1st year) vs. 42% (2nd year) [127,128]
		G2P[4]	[e]60.9% (1st year) vs. 63.4% (2nd year) [127,128]
		G12P[6]	[e]69.1% (1st year) vs. 69.7% (2nd year) [127,128]
		G12P[8]	[e]69.9% (1st year) vs. 69.2% (2nd year) [127,128]
		G9P[4]	[f]ND (1st year) vs. -343.5% (2nd year) [128]
		[g]Others	[h]41.4% (1st year) vs. 67.1% (2nd year) [127]
		All	[e]53.6% (1st year) vs. 55.1% (2nd year) [127,128]
	BRV-PV	[i]All	40.8% (1st year) vs. 38.2% (2nd year) [129]
**Middle East**			
[j]Israel	RV5	G1P[8]	64% (1D) vs. 89% (2Ds) vs. 78% (3Ds) vs. 78% (≥1D) vs. 81% (≥2Ds) [120]
**Africa**			
Botswana	RV1	G2P[4]	[k]33% (1D) vs. 59% (FD) vs. 53% (≥1D) [92]
Kenya	RV1	G1P[8[	60% [99]
		G2P[4]	[k]29% [99]
Malawi	RV1	G1	43.7% [145], 82% [130],70.7% [101,145]
		G2	53% [130], 45.9% [101], 6.2% [146]
		G8	48.4% [146]
		G9	58.3% [146]
		G12	53% [130], 51% [101], 49.5% [146]
		G1P[8]	82.1% [101]
		G2P[4]	34.9% [101]
		Non-G1	50.3% [146]
		P[4]	32.8% [101], 53.1% [146]
		P[6]	68.1% [101], 53.1% [146]

Country	*Vaccine*	*Genotypes*	*Vaccine effectiveness*
		P[8]	71% [101], 44.3% [146]
South Africa	RV1	G1	69.8% [145,146]
		G2	91.8% [146]
		G3	83.5% [146]
		G8	100.0% [146]
		G12	75.3% [146]
		Non-G1	85.9% [146]

D = dose; **AD** = any dose; **FD** = full doses; **ND** = not detected; **NC** = neighbourhood controls; **HC** = hospital controls; **NDC** = non-diarrhoea control; **TNC** = test-negative controls; **RAGE** = rotavirus acute gastroenteritis; **REST** = rotavirus efficacy and safety trial; **FES** = Finnish extension study

[a]The analysis was performed by comparing VE in REST group vs. REST+FES

[b]The analysis involved only the REST+FES

[c]95 – 100% prevalence of G2P[4] was observed between 2006 – 08 [119,147] and 82% prevalence between 2008 – 09 [83]

[d]Total VE against any severe ARG caused by homotypic vaccine strains in all age group = 48.1% and for heterotypic strains = 43.0%. There was no difference in all the calculated total VE of RV1 in Bangladesh when comparing vaccinated with unvaccinated groups, which suggests the poor performance of the RV1

[e]Progressing from the 1st to the 2nd year post-vaccination, there was no significant improvement in the efficacy of the vaccine against all the detected genotypes

[f]This genotype was not assessed in the first year but serendipitously discovered to be highly unequally distributed between the vaccine (9) and the placebo (1) groups in the second year of life. This has brought about a disconnected genotype-specific efficacy, and the authors concluded it was by chance since there was no biological reason for this imbalance

[g]Others are G0P[0], G1P[0], G1P[4], G1P[6], G2P[6], G9P[0], G9P[4], G9P[8], G12P[11] combined from the 1st and 2nd year of life after vaccination

[h]Evidence of increased vaccine efficacy against over multiple unusual genotypes within two years of follow-up

During the assessment of 116E in India, G9P[6] was not detected in any case of RVGE but shed decreasingly as doses increases in the 1st year of vaccination, but in the 2nd year it was highly unequally detected in 9 samples from the vaccine vs. 1 from the placebo

[i]All = vaccine genotypes (G1 – G4 and G9) as well as traces of G10 and G12 responsible for the majority (≈90%) of SRVGE cases

[j]In Israel, irrespective of the number of doses, RV5 produced almost equal effectiveness against G1P[8]

[k]Lower heterotypic protection.

References

1. Vesikari, T.; Isolauri, E.; D'Hondt, E.; Delem, A.; Andre, F.E. Increased "take" rate of oral rotavirus vaccine in infants after milk feeding. *Lancet* **1984**, *2*, 700, doi:10.1016/s0140-6736(84)91262-5.
2. Palmer, E.L.; Martin, M.L.; Murphy, F.A. Morphology and Stability of Infantile Gastroenteritis Virus - Comparison with Reovirus and Bluetongue Virus. *Journal of General Virology* **1977**, *35*, 403-414, doi:10.1099/0022-1317-35-3-403.
3. Estes, M.K.; Graham, D.Y.; Smith, E.M.; Gerba, C.P. Rotavirus stability and inactivation. *The Journal of general virology* **1979**, *43*, 403-409, doi:10.1099/0022-1317-43-2-403.
4. Vesikari, T.; Ruuska, T.; Bogaerts, H.; Delem, A.; Andre, F. Dose-response study of RIT4237 oral rotavirus vaccine in breast-fed and formula-fed infants. *Pediatr Infect Dis* **1985**, *4*, 622-625, doi:10.1097/00006454-198511000-00005.
5. Vesikari, T.; Ruuska, T.; Delem, A.; Andre, F.E. Oral rotavirus vaccination in breast- and bottle-fed infants aged 6 to 12 months. *Acta Paediatr Scand* **1986**, *75*, 573-578, doi:10.1111/j.1651-2227.1986.tb10253.x.
6. Vesikari, T.; Ruuska, T.; Delem, A.; André, F.E. Neonatal Rotavirus Vaccination with RIT4237 Bovine Rotavirus Vaccine: A Preliminary Report. *Pediatric Infectious Disease Journal* **1987**, *6*, 164-169, doi:10.1097/00006454-198702000-00005.
7. Maldonado, Y.; Hestvik, L.; Wilson, M.; Townsend, T.; O'Hare, J.; Wee, S.; Yolken, R. Safety and immunogenicity of bovine rotavirus vaccine RIT 4237 in 3-month-old infants. *The Journal of Pediatrics* **1986**, *109*, 931-935, doi:10.1016/S0022-3476(86)80271-2.
8. Bishop, R.F. Development of candidate rotavirus vaccines. *Vaccine* **1993**, *11*, 247-254, doi:10.1016/0264-410x(93)90025-s.
9. Hanlon, P.; Hanlon, L.; Marsh, V.; Byass, P.; Shenton, F.; Hassan-King, M.; Jobe, O.; Sillah, H.; Hayes, R.; M'Boge, B.H., et al. trial of an attenuated bovine rotavirus vaccine (RIT 4237) in Gambian infants. *Lancet* **1987**, *1*, 1342-1345, doi:10.1016/s0140-6736(87)90649-0.
10. De Mol, P.; Zissis, G.; Butzler, J.P.; Mutwewingabo, A.; Andre, F.E. Failure of live, attenuated oral rotavirus vaccine. *Lancet* **1986**, *2*, 108, doi:10.1016/s0140-6736(86)91643-0.
11. Rennels, M.B.; Losonsky, G.A.; Shindledecker, C.L.; Hughes, T.P.; Kapikian, A.Z.; Levine, M.M. Immunogenicity and reactogenicity of lowered doses of rhesus rotavirus vaccine strain MMU 18006 in young children. *Pediatr Infect Dis J* **1987**, *6*, 260-264, doi:10.1097/00006454-198703000-00010.
12. Losonsky, G.A.; Rennels, M.B.; Kapikian, A.Z.; Midthun, K.; Ferra, P.J.; Fortier, D.N.; Hoffman, K.M.; Baig, A.; Levine, M.M. Safety, infectivity, transmissibility and immunogenicity of rhesus rotavirus vaccine (MMU 18006) in infants. *Pediatr Infect Dis* **1986**, *5*, 25-29, doi:10.1097/00006454-198601000-00005.
13. Hoshino, Y.; Wyatt, R.G.; Greenberg, H.B.; Flores, J.; Kapikian, A.Z. Serotypic Similarity and Diversity of Rotaviruses of Mammalian and Avian Origin as Studied by Plaque-Reduction Neutralization. *Journal of Infectious Diseases* **1984**, *149*, 694-702, doi:10.1093/infdis/149.5.694.
14. Kapikian, A.Z.; Midthun, K.; Hoshino, Y.; Flores, J.; Wyatt, R.G.; Glass, R.I.; Askaa, J.; Nakagomi; Nakagomi, T.; Chanock, R.M., et al. Rhesus rotavirus: A candidate vaccine for prevention of human rotavirus disease. In *Vaccines 85: Molecular and Chemical Basis of Resistance to Parasitic, Bacterial and Viral Diseases*, Lerner, R.A., Chanock, R.M., Brown, F., Eds. Cold Spring Harbor Laboratory: 1985; pp. 357-367.
15. Anderson, E.L.; Belshe, R.B.; Bartram, J.; Crookshanks-Newman, F.; Chanock, R.M.; Kapikian, A.Z. Evaluation of rhesus rotavirus vaccine (MMU 18006) in infants and young children. *J Infect Dis* **1986**, *153*, 823-831, doi:10.1093/infdis/153.5.823.
16. Wright, P.F.; Tajima, T.; Thompson, J.; Kokubun, K.; Kapikian, A.; Karzon, D.T. Candidate Rotavirus Vaccine (Rhesus Rotavirus Strain) in Children - an Evaluation. *Pediatrics* **1987**, *80*, 473-480.

17. Rennels, M.B.; Losonsky, G.A.; Levine, M.M.; Kapikian, A.Z. Preliminary evaluation of the efficacy of rhesus rotavirus vaccine strain MMU 18006 in young children. *Pediatr Infect Dis* **1986**, *5*, 587-588, doi:10.1097/00006454-198609000-00019.
18. Christy, C.; Madore, H.P.; Pichichero, M.E.; Gala, C.; Pincus, P.; Vosefski, D.; Hoshino, Y.; Kapikian, A.; Dolin, R. Field trial of rhesus rotavirus vaccine in infants. *Pediatr Infect Dis J* **1988**, *7*, 645-650, doi:10.1097/00006454-198809000-00009.
19. Losonsky, G.A.; Rennels, M.B.; Lim, Y.; Krall, G.; Kapikian, A.Z.; Levine, M.M. Systemic and mucosal immune responses to rhesus rotavirus vaccine MMU 18006. *Pediatr Infect Dis J* **1988**, *7*, 388-393, doi:10.1097/00006454-198806000-00004.
20. Kapikian, A.Z.; Wyatt, R.G.; Levine, M.M.; Yolken, R.H.; VanKirk, D.H.; Dolin, R.; Greenberg, H.B.; Chanock, R.M. Oral administration of human rotavirus to volunteers: induction of illness and correlates of resistance. *J Infect Dis* **1983**, *147*, 95-106, doi:10.1093/infdis/147.1.95.
21. Perez-Schael, I.; Gonzalez, M.; Daoud, N.; Perez, M.; Soto, I.; Garcia, D.; Daoud, G.; Kapikian, A.Z.; Flores, J. Reactogenicity and antigenicity of the rhesus rotavirus vaccine in Venezuelan children. *J Infect Dis* **1987**, *155*, 334-338, doi:10.1093/infdis/155.2.334.
22. Vesikari, T.; Rautanen, T.; Varis, T.; Beards, G.M.; Kapikian, A.Z. Rhesus Rotavirus Candidate Vaccine - Clinical-Trial in Children Vaccinated between 2 and 5 Months of Age. *American Journal of Diseases of Children* **1990**, *144*, 285-289, doi:10.1001/archpedi.1990.02150270035021.
23. Flores, J.; Perez-Schael, I.; Gonzalez, M.; Garcia, D.; Perez, M.; Daoud, N.; Cunto, W.; Chanock, R.M.; Kapikian, A.Z. Protection against severe rotavirus diarrhoea by rhesus rotavirus vaccine in Venezuelan infants. *Lancet* **1987**, *1*, 882-884, doi:10.1016/s0140-6736(87)92858-3.
24. White, L.; Perez, I.; Perez, M.; Urbina, G.; Greenberg, H.; Kapikian, A.; Flores, J. Relative Frequency of Rotavirus Subgroup-1 and Subgroup-2 in Venezuelan Children with Gastroenteritis as Assayed with Monoclonal-Antibodies. *Journal of Clinical Microbiology* **1984**, *19*, 516-520, doi:10.1128/Jcm.19.4.516-520.1984.
25. Shaw, R.D.; Fong, K.J.; Losonsky, G.A.; Levine, M.M.; Maldonado, Y.; Yolken, R.; Flores, J.; Kapikian, A.Z.; Vo, P.T.; Greenberg, H.B. Epitope-specific immune responses to rotavirus vaccination. *Gastroenterology* **1987**, *93*, 941-950, doi:10.1016/0016-5085(87)90555-5.
26. Chiba, S.; Yokoyama, T.; Nakata, S.; Morita, Y.; Urasawa, T.; Taniguchi, K.; Urasawa, S.; Nakao, T. Protective effect of naturally acquired homotypic and heterotypic rotavirus antibodies. *Lancet* **1986**, *2*, 417-421, doi:10.1016/s0140-6736(86)92133-1.
27. Gothefors, L.; Wadell, G.; Juto, P.; Taniguchi, K.; Kapikian, A.Z.; Glass, R.I. Prolonged efficacy of rhesus rotavirus vaccine in Swedish children. *J Infect Dis* **1989**, *159*, 753-757, doi:10.1093/infdis/159.4.753.
28. Urasawa, S.; Urasawa, T.; Taniguchi, K.; Chiba, S. Serotype determination of human rotavirus isolates and antibody prevalence in pediatric population in Hokkaido, Japan. *Arch Virol* **1984**, *81*, 1-12, doi:10.1007/BF01309292.
29. Clark, H.F.; Furukawa, T.; Bell, L.M.; Offit, P.A.; Perrella, P.A.; Plotkin, S.A. Immune response of infants and children to low-passage bovine rotavirus (strain WC3). *Am J Dis Child* **1986**, *140*, 350-356, doi:10.1001/archpedi.1986.02140180084030.
30. Clark, H.F.; Borian, F.E.; Bell, L.M.; Modesto, K.; Gouvea, V.; Plotkin, S.A. Protective effect of WC3 vaccine against rotavirus diarrhea in infants during a predominantly serotype 1 rotavirus season. *J Infect Dis* **1988**, *158*, 570-587, doi:10.1093/infdis/158.3.570.
31. Kapikian, A.Z.; Chanock, R.M. Rotaviruses. In *Virology*, 2 ed.; Fields, B.N., Knipe, D.M., Eds. Raven Press Ltd: London, 1990; pp. 1353-1404.
32. Bernstein, D.I.; Smith, V.E.; Sander, D.S.; Pax, K.A.; Schiff, G.M.; Ward, R.L. Evaluation of WC3 rotavirus vaccine and correlates of protection in healthy infants. *J Infect Dis* **1990**, *162*, 1055-1062, doi:10.1093/infdis/162.5.1055.
33. Ward, R.L.; Sander, D.S.; Schiff, G.M.; Bernstein, D.I. Effect of vaccination on serotype-specific antibody responses in infants administered WC3 bovine rotavirus before or after a natural rotavirus infection. *J Infect Dis* **1990**, *162*, 1298-1303, doi:10.1093/infdis/162.6.1298.
34. Bishop, R.F.; Barnes, G.L.; Cipriani, E.; Lund, J.S. Clinical immunity after neonatal rotavirus infection. A prospective longitudinal study in young children. *N Engl J Med* **1983**, *309*, 72-76, doi:10.1056/NEJM198307143090203.

35. Barnes, G.L.; Lund, J.S.; Adams, L.; Mora, A.; Mitchell, S.V.; Caples, A.; Bishop, R.F. Phase 1 trial of a candidate rotavirus vaccine (RV3) derived from a human neonate. *J Paediatr Child Health* **1997**, *33*, 300-304, doi:10.1111/j.1440-1754.1997.tb01604.x.
36. Coulson, B.S.; Tursi, J.M.; Mcadam, W.J.; Bishop, R.F. Derivation of Neutralizing Monoclonal-Antibodies to Human Rotaviruses and Evidence That an Immunodominant Neutralization Site Is Shared between Serotypes-1 and Serotypes-3. *Virology* **1986**, *154*, 302-312, doi:10.1016/0042-6822(86)90456-3.
37. Barnes, G.L.; Lund, J.S.; Mitchell, S.V.; De Bruyn, L.; Piggford, L.; Smith, A.L.; Furmedge, J.; Masendycz, P.J.; Bugg, H.C.; Bogdanovic-Sakran, N., et al. Early phase II trial of human rotavirus vaccine candidate RV3. *Vaccine* **2002**, *20*, 2950-2956, doi:10.1016/s0264-410x(02)00235-9.
38. United States - Department of State - Bureau of Oceans - International Environmental and Scientific Affairs. *The United States-Japan Cooperative Medical Science Program: Fifth Five-year Report, 1986-1990*; Bureau of Oceans and International Environmental and Scientific Affairs: University of Minnesota, 1990; Vol. 9761, pp. 329.
39. Shigeo, M.; Shigeki, M.; Mitsuo, T.; Masami, H.; Sakae, I.; Ayako, H.; Konosuke, F. Cold-adaptation of human rotavirus. *Virus Research* **1987**, *7*, 273-280, doi:10.1016/0168-1702(87)90033-5.
40. Hoshino, Y.; Kapikian, A.Z. Rotavirus vaccine development for the prevention of severe diarrhea in infants and young children. *Trends Microbiol* **1994**, *2*, 242-249, doi:10.1016/0966-842x(94)90629-7.
41. Hoshino, Y.; Kapikian, A.Z.; Chanock, R.M. Attenuated human rotavirus vaccine. US7150984B2, 2006.
42. Vesikari, T.; Ruuska, T.; Green, K.Y.; Flores, J.; Kapikian, A.Z. Protective efficacy against serotype 1 rotavirus diarrhea by live oral rhesus-human reassortant rotavirus vaccines with human rotavirus VP7 serotype 1 or 2 specificity. *The Pediatric infectious disease journal* **1992**, *11*, 535-542, doi:10.1097/00006454-199207000-00006.
43. Flores, J.; Perez-Schael, I.; Blanco, M.; Vilar, M.; Garcia, D.; Perez, M.; Daoud, N.; Midthun, K.; Kapikian, A.Z. Reactions to and antigenicity of two human-rhesus rotavirus reassortant vaccine candidates of serotypes 1 and 2 in Venezuelan infants. *J Clin Microbiol* **1989**, *27*, 512-518, doi:0095-1137/89/030512-07$02.00/0.
44. Perez-Schael, I.; Blanco, M.; Vilar, M.; Garcia, D.; White, L.; Gonzalez, R.; Kapikian, A.Z.; Flores, J. Clinical studies of a quadrivalent rotavirus vaccine in Venezuelan infants. *J Clin Microbiol* **1990**, *28*, 553-558, doi:0095-1137/90/030553-06$02.00/0.
45. Paulke-Korinek, M.; Kollaritsch, H.; Aberle, S.W.; Zwazl, I.; Schmidle-Loss, B.; Vecsei, A.; Kundi, M. Sustained low hospitalization rates after four years of rotavirus mass vaccination in Austria. *Vaccine* **2013**, *31*, 2686-2691, doi:10.1016/j.vaccine.2013.04.001.
46. Paulke-Korinek, M.; Rendi-Wagner, P.; Kundi, M.; Kronik, R.; Kollaritsch, H. Universal mass vaccination against rotavirus gastroenteritis: impact on hospitalization rates in austrian children. *Pediatr Infect Dis J* **2010**, *29*, 319-323, doi:10.1097/INF.0b013e3181c18434.
47. Paulke-Korinek, M.; Kundi, M.; Rendi-Wagner, P.; de Martin, A.; Eder, G.; Schmidle-Loss, B.; Vecsei, A.; Kollaritsch, H. Herd immunity after two years of the universal mass vaccination program against rotavirus gastroenteritis in Austria. *Vaccine* **2011**, *29*, 2791-2796, doi:10.1016/j.vaccine.2011.01.104.
48. Zlamy, M.; Kofler, S.; Orth, D.; Wurzner, R.; Heinz-Erian, P.; Streng, A.; Prelog, M. The impact of Rotavirus mass vaccination on hospitalization rates, nosocomial Rotavirus gastroenteritis and secondary blood stream infections. *BMC Infect Dis* **2013**, *13*, 112, doi:10.1186/1471-2334-13-112.
49. Braeckman, T.; Van Herck, K.; Meyer, N.; Pircon, J.Y.; Soriano-Gabarro, M.; Heylen, E.; Zeller, M.; Azou, M.; Capiau, H.; De Koster, J., et al. Effectiveness of rotavirus vaccination in prevention of hospital admissions for rotavirus gastroenteritis among young children in Belgium: case-control study. *BMJ* **2012**, *345*, e4752, doi:10.1136/bmj.e4752.
50. Zeller, M.; Rahman, M.; Heylen, E.; De Coster, S.; De Vos, S.; Arijs, I.; Novo, L.; Verstappen, N.; Van Ransta, M.; Matthijnssens, J. Rotavirus incidence and genotype distribution before and after national rotavirus vaccine introduction in Belgium. *Vaccine* **2010**, *28*, 7507-7513, doi:10.1016/j.vaccine.2010.09.004.

51. Braeckman, T.; Van Herck, K.; Raes, M.; Vergison, A.; Sabbe, M.; Van Damme, P. Rotavirus vaccines in Belgium: policy and impact. *Pediatr Infect Dis J* **2011**, *30*, S21-24, doi:10.1097/INF.0b013e3181fefc51.
52. Raes, M.; Strens, D.; Vergison, A.; Verghote, M.; Standaert, B. Reduction in pediatric rotavirus-related hospitalizations after universal rotavirus vaccination in Belgium. *Pediatr Infect Dis J* **2011**, *30*, e120-125, doi:10.1097/INF.0b013e318214b811.
53. Hanquet, G.; Ducoffre, G.; Vergison, A.; Neels, P.; Sabbe, M.; Van Damme, P.; Van Herck, K. Impact of rotavirus vaccination on laboratory confirmed cases in Belgium. *Vaccine* **2011**, *29*, 4698-4703, doi:10.1016/j.vaccine.2011.04.098.
54. Vesikari, T.; Uhari, M.; Renko, M.; Hemming, M.; Salminen, M.; Torcel-Pagnon, L.; Bricout, H.; Simondon, F. Impact and Effectiveness of RotaTeq (R) Vaccine Based on 3 Years of Surveillance Following Introduction of a Rotavirus Immunization Program in Finland. *Pediatric Infectious Disease Journal* **2013**, *32*, 1365-1373, doi:10.1097/Inf.0000000000000086.
55. Leino, T.; Ollgren, J.; Salo, H.; Tiihonen, P.; Kilpi, T. First year experience of rotavirus immunisation programme in Finland. *Vaccine* **2012**, *31*, 176-182, doi:10.1016/j.vaccine.2012.10.068.
56. Hemming, M.; Räsänen, S.; Huhti, L.; Paloniemi, M.; Salminen, M.; Vesikari, T. Major reduction of rotavirus, but not norovirus, gastroenteritis in children seen in hospital after the introduction of RotaTeq vaccine into the National Immunization Programme in Finland. *European journal of pediatrics* **2013**, *172*, 739-746, doi:10.1007/s00431-013-1945-3.
57. Gagneur, A.; Nowak, E.; Lemaitre, T.; Segura, J.F.; Delaperriere, N.; Abalea, L.; Poulhazan, E.; Jossens, A.; Auzanneau, L.; Tran, A., et al. Impact of rotavirus vaccination on hospitalizations for rotavirus diarrhea: the IVANHOE study. *Vaccine* **2011**, *29*, 3753-3759, doi:10.1016/j.vaccine.2011.03.035.
58. Dudareva-Vizule, S.; Koch, J.; An der Heiden, M.; Oberle, D.; Keller-Stanislawski, B.; Wichmann, O. Impact of rotavirus vaccination in regions with low and moderate vaccine uptake in Germany. *Hum Vaccin Immunother* **2012**, *8*, 1407-1415, doi:10.4161/hv.21593.
59. Adlhoch, C.; Hoehne, M.; Littmann, M.; Marques, A.M.; Lerche, A.; Dehnert, M.; Eckmanns, T.; Wichmann, O.; Koch, J. Rotavirus vaccine effectiveness and case-control study on risk factors for breakthrough infections in Germany, 2010-2011. *Pediatr Infect Dis J* **2013**, *32*, e82-89, doi:10.1097/INF.0b013e3182720b71.
60. Gheorghita, S.; Birca, L.; Donos, A.; Wasley, A.; Birca, I.; Cojocaru, R.; Melnick, A.; Ciobanu, S.; Mosina, L.; Cortese, M.M., et al. Impact of Rotavirus Vaccine Introduction and Vaccine Effectiveness in the Republic of Moldova. *Clin Infect Dis* **2016**, *62 Suppl 2*, S140-146, doi:10.1093/cid/civ1209.
61. Rodrigues, F.; Iturriza-Gomara, M.; Marlow, R.; Gray, J.; Nawaz, S.; Januario, L.; Finn, A. The evolving epidemiology of rotavirus gastroenteritis in central Portugal with modest vaccine coverage. *J Clin Virol* **2013**, *56*, 129-134, doi:10.1016/j.jcv.2012.10.016.
62. Marlow, R.; Ferreira, M.; Cordeiro, E.; Trotter, C.; Januario, L.; Finn, A.; Rodrigues, F. Case Control Study of Rotavirus Vaccine Effectiveness in Portugal During 6 Years of Private Market Use. *Pediatric Infectious Disease Journal* **2015**, *34*, 509-512, doi:10.1097/Inf.0000000000000647.
63. Martinon-Torres, F.; Bouzon Alejandro, M.; Redondo Collazo, L.; Sanchez Lastres, J.M.; Pertega Diaz, S.; Seoane Pillado, M.T.; Martinon Sanchez, J.M.; team, R.r. Effectiveness of rotavirus vaccination in Spain. *Hum Vaccin* **2011**, *7*, 757-761, doi:10.4161/hv.7.7.15576.
64. Castilla, J.; Beristain, X.; Martinez-Artola, V.; Navascues, A.; Cenoz, M.G.; Alvarez, N.; Polo, I.; Mazon, A.; Gil-Setas, A.; Barricarte, A. Effectiveness of rotavirus vaccines in preventing cases and hospitalizations due to rotavirus gastroenteritis in Navarre, Spain. *Vaccine* **2012**, *30*, 539-543, doi:10.1016/j.vaccine.2011.11.071.
65. Gil-Prieto, R.; Gonzalez-Escalada, A.; Alvaro-Meca, A.; Garcia-Garcia, L.; San-Martin, M.; Gonzalez-Lopez, A.; Gil-de-Miguel, A. Impact of non-routine rotavirus vaccination on hospitalizations for diarrhoea and rotavirus infections in Spain. *Vaccine* **2013**, *31*, 5000-5004, doi:10.1016/j.vaccine.2013.05.109.

66. Martinón-Torres, F.; Martinón-Torres, N.; Alejandro, M.B.; Collazo, L.R.; Pértega-Díaz, S.; Seoane-Pillado, M.T.; Viñas, J.A.; San-Martín, M. Acute gastroenteritis hospitalizations among children aged <5 years before and after introduction of rotavirus vaccines: a hospital-based surveillance study in Galicia, Spain. *Human vaccines & immunotherapeutics* **2012**, *8*, 946-952, doi:10.4161/hv.20178.
67. Doll, M.K.; Buckeridge, D.L.; Morrison, K.T.; Gagneur, A.; Tapiero, B.; Charest, H.; Quach, C. Effectiveness of monovalent rotavirus vaccine in a high-income, predominant-use setting. *Vaccine* **2015**, *33*, 7307-7314, doi:10.1016/j.vaccine.2015.10.118.
68. Cortese, M.M.; Immergluck, L.C.; Held, M.; Jain, S.; Chan, T.; Grizas, A.P.; Khizer, S.; Barrett, C.; Quaye, O.; Mijatovic-Rustempasic, S., et al. Effectiveness of monovalent and pentavalent rotavirus vaccine. *Pediatrics* **2013**, *132*, e25-33, doi:10.1542/peds.2012-3804.
69. Payne, D.C.; Boom, J.A.; Staat, M.A.; Edwards, K.M.; Szilagyi, P.G.; Klein, E.J.; Selvarangan, R.; Azimi, P.H.; Harrison, C.; Moffatt, M. Effectiveness of pentavalent and monovalent rotavirus vaccines in concurrent use among US children <5 years of age, 2009–2011. *Clinical infectious diseases* **2013**, *57*, 13-20, doi:10.1093/cid/cit164.
70. Payne, D.C.; Selvarangan, R.; Azimi, P.H.; Boom, J.A.; Englund, J.A.; Staat, M.A.; Halasa, N.B.; Weinberg, G.A.; Szilagyi, P.G.; Chappell, J., et al. Long-term Consistency in Rotavirus Vaccine Protection: RV5 and RV1 Vaccine Effectiveness in US Children, 2012-2013. *Clin Infect Dis* **2015**, *61*, 1792-1799, doi:10.1093/cid/civ872.
71. Cortese, M.M.; Leblanc, J.; White, K.E.; Jerris, R.C.; Stinchfield, P.; Preston, K.L.; Meek, J.; Odofin, L.; Khizer, S.; Miller, C.A., et al. Leveraging state immunization information systems to measure the effectiveness of rotavirus vaccine. *Pediatrics* **2011**, *128*, e1474-1481, doi:10.1542/peds.2011-1006.
72. Staat, M.A.; Payne, D.C.; Donauer, S.; Weinberg, G.A.; Edwards, K.M.; Szilagyi, P.G.; Griffin, M.R.; Hall, C.B.; Curns, A.T.; Gentsch, J.R., et al. Effectiveness of pentavalent rotavirus vaccine against severe disease. *Pediatrics* **2011**, *128*, e267-275, doi:10.1542/peds.2010-3722.
73. Paternina-Caicedo, A.; Parashar, U.D.; Alvis-Guzmán, N.; De Oliveira, L.H.; Castaño-Zuluaga, A.; Cotes-Cantillo, K.; Gamboa-Garay, O.; Coronell-Rodríguez, W.; De la Hoz-Restrepo, F. Effect of rotavirus vaccine on childhood diarrhea mortality in five Latin American countries. *Vaccine* **2015**, *33*, 3923-3928, doi:10.1016/j.vaccine.2015.06.058.
74. de Palma, O.; Cruz, L.; Ramos, H.; de Baires, A.; Villatoro, N.; Pastor, D.; de Oliveira, L.H.; Kerin, T.; Bowen, M.; Gentsch, J., et al. Effectiveness of rotavirus vaccination against childhood diarrhoea in El Salvador: case-control study. *BMJ* **2010**, *340*, c2825, doi:10.1136/bmj.c2825.
75. Gastanaduy, P.A.; Contreras-Roldan, I.; Bernart, C.; Lopez, B.; Benoit, S.R.; Xuya, M.; Munoz, F.; Desai, R.; Quaye, O.; Tam, K.I., et al. Effectiveness of Monovalent and Pentavalent Rotavirus Vaccines in Guatemala. *Clin Infect Dis* **2016**, *62 Suppl 2*, S121-126, doi:10.1093/cid/civ1208.
76. Richardson, V.; Parashar, U.; Patel, M. Childhood diarrhea deaths after rotavirus vaccination in Mexico. *N Engl J Med* **2011**, *365*, 772-773, doi:10.1056/NEJMc1100062.
77. Quintanar-Solares, M.; Yen, C.; Richardson, V.; Esparza-Aguilar, M.; Parashar, U.D.; Patel, M.M. Impact of Rotavirus Vaccination on Diarrhea-related Hospitalizations Among Children <5 Years of Age in Mexico. *The Pediatric Infectious Disease Journal* **2011**, *30*, S11-S15, doi:10.1097/INF.0b013e3181fefb32.
78. Patel, M.; Pedreira, C.; De Oliveira, L.H.; Tate, J.; Orozco, M.; Mercado, J.; Gonzalez, A.; Malespin, O.; Amador, J.J.; Umana, J., et al. Association Between Pentavalent Rotavirus Vaccine and Severe Rotavirus Diarrhea Among Children in Nicaragua. *Jama-Journal of the American Medical Association* **2009**, *301*, 2243-2251, doi:10.1001/jama.2009.756.
79. Becker-Dreps, S.; Paniagua, M.; Dominik, R.; Cao, H.; Shah, N.K.; Morgan, D.R.; Moreno, G.; Espinoza, F. Changes in childhood diarrhea incidence in nicaragua following 3 years of universal infant rotavirus immunization. *Pediatr Infect Dis J* **2011**, *30*, 243-247, doi:10.1097/INF.0b013e3181f87ffe.
80. Patel, M.; Pedreira, C.; De Oliveira, L.H.; Umana, J.; Tate, J.; Lopman, B.; Sanchez, E.; Reyes, M.; Mercado, J.; Gonzalez, A., et al. Duration of protection of pentavalent rotavirus vaccination in Nicaragua. *Pediatrics* **2012**, *130*, e365-372, doi:10.1542/peds.2011-3478.

81. Pringle, K.D.; Patzi, M.; Tate, J.E.; Rojas, V.I.; Patel, M.; Jordan, L.I.; Montesano, R.; Zarate, A.; De Oliveira, L.; Parashar, U. Sustained Effectiveness of Rotavirus Vaccine Against Very Severe Rotavirus Disease Through the Second Year of Life, Bolivia 2013-2014. *Clinical Infectious Diseases* **2016**, *62*, S115-S120, doi:10.1093/cid/civ1026.
82. Patel, M.M.; Patzi, M.; Pastor, D.; Nina, A.; Roca, Y.; Alvarez, L.; Iniguez, V.; Rivera, R.; Tam, K.I.; Quaye, O., et al. Effectiveness of monovalent rotavirus vaccine in Bolivia: case-control study. *BMJ* **2013**, *346*, f3726, doi:10.1136/bmj.f3726.
83. Justino, M.C.; Linhares, A.C.; Lanzieri, T.M.; Miranda, Y.; Mascarenhas, J.D.; Abreu, E.; Guerra, S.F.; Oliveira, A.S.; da Silva, V.B.; Sanchez, N., et al. Effectiveness of the monovalent G1P[8] human rotavirus vaccine against hospitalization for severe G2P[4] rotavirus gastroenteritis in Belem, Brazil. *Pediatr Infect Dis J* **2011**, *30*, 396-401, doi:10.1097/INF.0b013e3182055cc2.
84. Linhares, A.C.; Justino, M.C. Rotavirus vaccination in Brazil: effectiveness and health impact seven years post-introduction. *Expert Rev Vaccines* **2014**, *13*, 43-57, doi:10.1586/14760584.2014.861746.
85. Justino, M.C.A.; Brasil, P.; Abreu, E.; Miranda, Y.; Mascarenhas, J.D.P.; Guerra, S.F.S.; Linhares, A.C. Clinical Severity and Rotavirus Vaccination among Children Hospitalized for Acute Gastroenteritis in Belem, Northern Brazil. *Journal of Tropical Pediatrics* **2016**, *62*, 276-281, doi:10.1093/tropej/fmv098.
86. Cotes-Cantillo, K.; Paternina-Caicedo, A.; Coronell-Rodriguez, W.; Alvis-Guzman, N.; Parashar, U.D.; Patel, M.; De la Hoz-Restrepo, F. Effectiveness of the monovalent rotavirus vaccine in Colombia: a case-control study. *Vaccine* **2014**, *32*, 3035-3040, doi:10.1016/j.vaccine.2014.03.064.
87. De la Hoz, F.; Alvis, N.; Narvaez, J.; Cediel, N.; Gamboa, O.; Velandia, M. Potential epidemiological and economical impact of two rotavirus vaccines in Colombia. *Vaccine* **2010**, *28*, 3856-3864, doi:10.1016/j.vaccine.2010.03.004.
88. Sahakyan, G.; Grigoryan, S.; Wasley, A.; Mosina, L.; Sargsyan, S.; Asoyan, A.; Gevorgyan, Z.; Kocharyan, K.; Avagyan, T.; Lopman, B., et al. Impact and Effectiveness of Monovalent Rotavirus Vaccine in Armenian Children. *Clin Infect Dis* **2016**, *62 Suppl 2*, S147-154, doi:10.1093/cid/ciw045.
89. Zaman, K.; Sack, D.A.; Neuzil, K.M.; Yunus, M.; Moulton, L.H.; Sugimoto, J.D.; Fleming, J.A.; Hossain, I.; Arifeen, S.E.; Azim, T., et al. Effectiveness of a live oral human rotavirus vaccine after programmatic introduction in Bangladesh: A cluster-randomized trial. *PLoS Med* **2017**, *14*, e1002282, doi:10.1371/journal.pmed.1002282.
90. Muhsen, K.; Chodick, G.; Goren, S.; Shalev, V.; Cohen, D. The uptake of rotavirus vaccine and its effectiveness in preventing acute gastroenteritis in the community. *Vaccine* **2010**, *29*, 91-94.
91. Muhsen, K.; Shulman, L.; Kasem, E.; Rubinstein, U.; Shachter, J.; Kremer, A.; Goren, S.; Zilberstein, I.; Chodick, G.; Ephros, M., et al. Effectiveness of rotavirus vaccines for prevention of rotavirus gastroenteritis-associated hospitalizations in Israel: a case-control study. *Hum Vaccin* **2010**, *6*, 450-454, doi:10.4161/hv.6.6.11759.
92. Gastanaduy, P.A.; Steenhoff, A.P.; Mokomane, M.; Esona, M.D.; Bowen, M.D.; Jibril, H.; Pernica, J.M.; Mazhani, L.; Smieja, M.; Tate, J.E., et al. Effectiveness of Monovalent Rotavirus Vaccine After Programmatic Implementation in Botswana: A Multisite Prospective Case-Control Study. *Clin Infect Dis* **2016**, *62 Suppl 2*, S161-167, doi:10.1093/cid/civ1207.
93. Mokomane, M.; Esona, M.D.; Bowen, M.D.; Tate, J.E.; Steenhoff, A.P.; Lechiile, K.; Gaseitsiwe, S.; Seheri, L.M.; Magagula, N.B.; Weldegebriel, G., et al. Diversity of Rotavirus Strains Circulating in Botswana before and after introduction of the Monovalent Rotavirus Vaccine. *Vaccine* **2019**, *37*, 6324-6328, doi:10.1016/j.vaccine.2019.09.022.
94. Enane, L.A.; Gastañaduy, P.A.; Goldfarb, D.M.; Pernica, J.M.; Mokomane, M.; Moorad, B.; Masole, L.; Tate, J.E.; Parashar, U.D.; Steenhoff, A.P. Impact of Rotavirus Vaccination on Hospitalizations and Deaths From Childhood Gastroenteritis in Botswana. *Clinical Infectious Diseases* **2016**, *62*, S168-S174, doi:10.1093/cid/civ1210.

95. Bonkoungou, I.J.O.; Aliabadi, N.; Leshem, E.; Kam, M.; Nezien, D.; Drabo, M.K.; Nikiema, M.; Ouedraogo, B.; Medah, I.; Konate, S., et al. Impact and effectiveness of pentavalent rotavirus vaccine in children <5years of age in Burkina Faso. *Vaccine* **2018**, *36*, 7170-7178, doi:10.1016/j.vaccine.2017.12.056.
96. Sanneh, B.; Papa Sey, A.; Shah, M.; Tate, J.; Sonko, M.; Jagne, S.; Jarju, M.; Sowe, D.; Taal, M.; Cohen, A., et al. Impact of pentavalent rotavirus vaccine against severe rotavirus diarrhoea in The Gambia. *Vaccine* **2018**, *36*, 7179-7184, doi:10.1016/j.vaccine.2018.02.091.
97. Armah, G.; Pringle, K.; Enweronu-Laryea, C.C.; Ansong, D.; Mwenda, J.M.; Diamenu, S.K.; Narh, C.; Lartey, B.; Binka, F.; Grytdal, S., et al. Impact and Effectiveness of Monovalent Rotavirus Vaccine Against Severe Rotavirus Diarrhea in Ghana. *Clinical Infectious Diseases* **2016**, *62*, S200-S207, doi:10.1093/cid/ciw014.
98. Armah, G.E.; Sow, S.O.; Breiman, R.F.; Dallas, M.J.; Tapia, M.D.; Feikin, D.R.; Binka, F.N.; Steele, A.D.; Laserson, K.F.; Ansah, N.A., et al. Efficacy of pentavalent rotavirus vaccine against severe rotavirus gastroenteritis in infants in developing countries in sub-Saharan Africa: a randomised, double-blind, placebo-controlled trial. *Lancet* **2010**, *376*, 606-614, doi:10.1016/S0140-6736(10)60889-6.
99. Khagayi, S.; Omore, R.; Otieno, G.; Ogwel, B.; Ochieng, J.; Juma, J.; Apondi, E.; Bigogo, G.; Onyango, C.; Ngama, M., et al. Effectiveness of monovalent rotavirus vaccine against hospitalization with acute rotavirus gastroenteritis in Kenyan children. *Clinical infectious diseases : an official publication of the Infectious Diseases Society of America* **2019**, 10.1093/cid/ciz664, doi:10.1093/cid/ciz664.
100. Rahajamanana, V.L.; Raboba, J.L.; Rakotozanany, A.; Razafindraibe, N.J.; Andriatahirintsoa, E.J.P.R.; Razafindrakoto, A.C.; Mioramalala, S.A.; Razaiarimanga, C.; Weldegebriel, G.G.; Burnett, E., et al. Impact of rotavirus vaccine on all-cause diarrhea and rotavirus hospitalizations in Madagascar. *Vaccine* **2018**, *36*, 7198-7204, doi:10.1016/j.vaccine.2017.08.091.
101. Bar-Zeev, N.; Jere, K.C.; Bennett, A.; Pollock, L.; Tate, J.E.; Nakagomi, O.; Iturriza-Gomara, M.; Costello, A.; Mwansambo, C.; Parashar, U.D., et al. Population Impact and Effectiveness of Monovalent Rotavirus Vaccination in Urban Malawian Children 3 Years After Vaccine Introduction: Ecological and Case-Control Analyses. *Clin Infect Dis* **2016**, *62 Suppl 2*, S213-219, doi:10.1093/cid/civ1183.
102. de Deus, N.; Chilaule, J.J.; Cassocera, M.; Bambo, M.; Langa, J.S.; Sitoe, E.; Chissaque, A.; Anapakala, E.; Sambo, J.; Guimaraes, E.L., et al. Early impact of rotavirus vaccination in children less than five years of age in Mozambique. *Vaccine* **2018**, *36*, 7205-7209, doi:10.1016/j.vaccine.2017.10.060.
103. Ngabo, F.; Tate, J.E.; Gatera, M.; Rugambwa, C.; Donnen, P.; Lepage, P.; Mwenda, J.M.; Binagwaho, A.; Parashar, U.D. Effect of pentavalent rotavirus vaccine introduction on hospital admissions for diarrhoea and rotavirus in children in Rwanda: a time-series analysis. *Lancet Glob Health* **2016**, *4*, e129-136, doi:10.1016/S2214-109X(15)00270-3.
104. Diop, A.; Thiongane, A.; Mwenda, J.M.; Aliabadi, N.; Sonko, M.A.; Diallo, A.; Ndoye, B.; Faye, P.M.; Ba, I.D.; Parashar, U.D., et al. Impact of rotavirus vaccine on acute gastroenteritis in children under 5 years in Senegal: Experience of sentinel site of the Albert Royer Children's Hospital in Dakar. *Vaccine* **2018**, *36*, 7192-7197, doi:10.1016/j.vaccine.2017.10.061.
105. Groome, M.J.; Page, N.; Cortese, M.M.; Moyes, J.; Zar, H.J.; Kapongo, C.N.; Mulligan, C.; Diedericks, R.; Cohen, C.; Fleming, J.A., et al. Effectiveness of monovalent human rotavirus vaccine against admission to hospital for acute rotavirus diarrhoea in South African children: a case-control study. *Lancet Infect Dis* **2014**, *14*, 1096-1104, doi:10.1016/S1473-3099(14)70940-5.
106. Maphalala, G.; Phungwayo, N.; Masona, G.; Lukhele, N.; Tsegaye, G.; Dube, N.; Sindisiwe, D.; Khumalo, L.; Daniel, F.; Katsande, R., et al. Early impact of rotavirus vaccine in under 5year old children hospitalized due to diarrhea, Swaziland. *Vaccine* **2018**, *36*, 7210-7214, doi:10.1016/j.vaccine.2017.07.072.
107. Abeid, K.A.; Jani, B.; Cortese, M.M.; Kamugisha, C.; Mwenda, J.M.; Pandu, A.S.; Msaada, K.A.; Mohamed, A.S.; Khamis, A.U.; Parashar, U.D., et al. Monovalent Rotavirus Vaccine Effectiveness and Impact on Rotavirus Hospitalizations in Zanzibar, Tanzania: Data From the First 3 Years After Introduction. *The Journal of Infectious Diseases* **2016**, *215*, 183-191, doi:10.1093/infdis/jiw524.

108. Tsolenyanu, E.; Djadou, K.E.; Fiawoo, M.; Akolly, D.A.E.; Mwenda, J.M.; Leshem, E.; Tate, J.E.; Aliabadi, N.; Koudema, W.; Guedenon, K.M., et al. Evidence of the impact of monovalent rotavirus vaccine on childhood acute gastroenteritis hospitalization in Togo. *Vaccine* **2018**, *36*, 7185-7191, doi:10.1016/j.vaccine.2018.01.058.
109. Beres, L.K.; Tate, J.E.; Njobvu, L.; Chibwe, B.; Rudd, C.; Guffey, M.B.; Stringer, J.S.; Parashar, U.D.; Chilengi, R. A Preliminary Assessment of Rotavirus Vaccine Effectiveness in Zambia. *Clin Infect Dis* **2016**, *62 Suppl 2*, S175-182, doi:10.1093/cid/civ1206.
110. Mpabalwani, E.M.; Simwaka, C.J.; Mwenda, J.M.; Mubanga, C.P.; Monze, M.; Matapo, B.; Parashar, U.D.; Tate, J.E. Impact of Rotavirus Vaccination on Diarrheal Hospitalizations in Children Aged <5 Years in Lusaka, Zambia. *Clin Infect Dis* **2016**, *62 Suppl 2*, S183-187, doi:10.1093/cid/civ1027.
111. Mukaratirwa, A.; Berejena, C.; Nziramasanga, P.; Ticklay, I.; Gonah, A.; Nathoo, K.; Manangazira, P.; Mangwanya, D.; Marembo, J.; Mwenda, J.M., et al. Distribution of rotavirus genotypes associated with acute diarrhoea in Zimbabwean children less than five years old before and after rotavirus vaccine introduction. *Vaccine* **2018**, *36*, 7248-7255, doi:10.1016/j.vaccine.2018.03.069.
112. Mujuru, H.A.; Yen, C.; Nathoo, K.J.; Gonah, N.A.; Ticklay, I.; Mukaratirwa, A.; Berejena, C.; Tapfumanei, O.; Chindedza, K.; Rupfutse, M., et al. Reduction in Diarrhea- and Rotavirus-related Healthcare Visits Among Children <5 Years of Age After National Rotavirus Vaccine Introduction in Zimbabwe. *Pediatr Infect Dis J* **2017**, *36*, 995-999, doi:10.1097/INF.0000000000001648.
113. Mujuru, H.A.; Burnett, E.; Nathoo, K.J.; Ticklay, I.; Gonah, N.A.; Mukaratirwa, A.; Berejena, C.; Manangazira, P.; Rupfutse, M.; Weldegebriel, G.G., et al. Monovalent Rotavirus Vaccine Effectiveness Against Rotavirus Hospitalizations Among Children in Zimbabwe. *Clinical Infectious Diseases* **2019**, *69*, 1339-1344, doi:10.1093/cid/ciy1096.
114. Jonesteller, C.L.; Burnett, E.; Yen, C.; Tate, J.E.; Parashar, U.D. Effectiveness of Rotavirus Vaccination: A Systematic Review of the First Decade of Global Postlicensure Data, 2006-2016. *Clin Infect Dis* **2017**, *65*, 840-850, doi:10.1093/cid/cix369.
115. Isanaka, S.; Guindo, O.; Langendorf, C.; Matar Seck, A.; Plikaytis, B.D.; Sayinzoga-Makombe, N.; McNeal, M.M.; Meyer, N.; Adehossi, E.; Djibo, A., et al. Efficacy of a Low-Cost, Heat-Stable Oral Rotavirus Vaccine in Niger. *N Engl J Med* **2017**, *376*, 1121-1130, doi:10.1056/NEJMoa1609462.
116. Vesikari, T.; Karvonen, A.; Ferrante, S.A.; Kuter, B.J.; Ciarlet, M. Sustained efficacy of the pentavalent rotavirus vaccine, RV5, up to 3.1 years following the last dose of vaccine. *Pediatr Infect Dis J* **2010**, *29*, 957-963, doi:10.1097/INF.0b013e3181e28e6e.
117. Boom, J.A.; Tate, J.E.; Sahni, L.C.; Rench, M.A.; Quaye, O.; Mijatovic-Rustempasic, S.; Patel, M.M.; Baker, C.J.; Parashar, U.D. Sustained Protection from Pentavalent Rotavirus Vaccination during the Second Year of Life at a Large, Urban United States Pediatric Hospital. *Pediatric Infectious Disease Journal* **2010**, *29*, 1133-1135, doi:10.1097/INF.0b013e3181ed18ab.
118. Mast, T.C.; Khawaja, S.; Espinoza, F.; Paniagua, M.; Del Carmen, L.P.; Cardellino, A.; Sanchez, E. Case-control study of the effectiveness of vaccination with pentavalent rotavirus vaccine in Nicaragua. *Pediatr Infect Dis J* **2011**, *30*, e209-215, doi:10.1097/INF.0b013e31822a8527.
119. Correia, J.B.; Patel, M.M.; Nakagomi, O.; Montenegro, F.M.; Germano, E.M.; Correia, N.B.; Cuevas, L.E.; Parashar, U.D.; Cunliffe, N.A.; Nakagomi, T. Effectiveness of monovalent rotavirus vaccine (Rotarix) against severe diarrhea caused by serotypically unrelated G2P[4] strains in Brazil. *J Infect Dis* **2010**, *201*, 363-369, doi:10.1086/649843.
120. Leshem, E.; Givon-Lavi, N.; Tate, J.E.; Greenberg, D.; Parashar, U.D.; Dagan, R. Real-World Effectiveness of Pentavalent Rotavirus Vaccine Among Bedouin and Jewish Children in Southern Israel. *Clinical Infectious Diseases* **2016**, *62*, S155-S160, doi:10.1093/cid/civ1012.
121. Tate, J.E.; Ngabo, F.; Donnen, P.; Gatera, M.; Uwimana, J.; Rugambwa, C.; Mwenda, J.M.; Parashar, U.D. Effectiveness of Pentavalent Rotavirus Vaccine Under Conditions of Routine Use in Rwanda. *Clinical Infectious Diseases* **2016**, *62*, S208-S212, doi:10.1093/cid/civ1016.

122. Pérez-Vilar, S.; Díez-Domingo, J.; López-Lacort, M.; Martínez-Úbeda, S.; Martinez-Beneito, M.A. Effectiveness of rotavirus vaccines, licensed but not funded, against rotavirus hospitalizations in the Valencia Region, Spain. *BMC infectious diseases* **2015**, *15*, 92.
123. Boom, J.A.; Tate, J.E.; Sahni, L.C.; Rench, M.A.; Hull, J.J.; Gentsch, J.R.; Patel, M.M.; Baker, C.J.; Parashar, U.D. Effectiveness of pentavalent rotavirus vaccine in a large urban population in the United States. *Pediatrics* **2010**, *125*, e199-207, doi:10.1542/peds.2009-1021.
124. Donauer, S.; Payne, D.C.; Edwards, K.M.; Szilagyi, P.G.; Hornung, R.W.; Weinberg, G.A.; Chappell, J.; Hall, C.B.; Parashar, U.D.; Staat, M.A. Determining the effectiveness of the pentavalent rotavirus vaccine against rotavirus hospitalizations and emergency department visits using two study designs. *Vaccine* **2013**, *31*, 2692-2697, doi:10.1016/j.vaccine.2013.03.072.
125. Yen, C.; Figueroa, J.R.; Uribe, E.S.; Carmen-Hernandez, L.D.; Tate, J.E.; Parashar, U.D.; Patel, M.M.; Richardson Lopez-Collado, V. Monovalent rotavirus vaccine provides protection against an emerging fully heterotypic G9P[4] rotavirus strain in Mexico. *J Infect Dis* **2011**, *204*, 783-786, doi:10.1093/infdis/jir390.
126. Ichihara, M.Y.; Rodrigues, L.C.; Teles Santos, C.A.; Teixeira Mda, G.; De Jesus, S.R.; Alvim De Matos, S.M.; Gagliardi Leite, J.P.; Barreto, M.L. Effectiveness of rotavirus vaccine against hospitalized rotavirus diarrhea: A case-control study. *Vaccine* **2014**, *32*, 2740-2747, doi:10.1016/j.vaccine.2014.01.007.
127. Bhandari, N.; Rongsen-Chandola, T.; Bavdekar, A.; John, J.; Antony, K.; Taneja, S.; Goyal, N.; Kawade, A.; Kang, G.; Rathore, S.S., et al. Efficacy of a monovalent human-bovine (116E) rotavirus vaccine in Indian infants: a randomised, double-blind, placebo-controlled trial. *Lancet* **2014**, *383*, 2136-2143, doi:10.1016/S0140-6736(13)62630-6.
128. Bhandari, N.; Rongsen-Chandola, T.; Bavdekar, A.; John, J.; Antony, K.; Taneja, S.; Goyal, N.; Kawade, A.; Kang, G.; Rathore, S.S., et al. Efficacy of a monovalent human-bovine (116E) rotavirus vaccine in Indian children in the second year of life. *Vaccine* **2014**, *32 Suppl 1*, A110-116, doi:10.1016/j.vaccine.2014.04.079.
129. Kulkarni, P.S.; Desai, S.; Tewari, T.; Kawade, A.; Goyal, N.; Garg, B.S.; Kumar, D.; Kanungo, S.; Kamat, V.; Kang, G., et al. A randomized Phase III clinical trial to assess the efficacy of a bovine-human reassortant pentavalent rotavirus vaccine in Indian infants. *Vaccine* **2017**, *35*, 6228-6237, doi:10.1016/j.vaccine.2017.09.014.
130. Bar-Zeev, N.; Kapanda, L.; Tate, J.E.; Jere, K.C.; Iturriza-Gomara, M.; Nakagomi, O.; Mwansambo, C.; Costello, A.; Parashar, U.D.; Heyderman, R.S., et al. Effectiveness of a monovalent rotavirus vaccine in infants in Malawi after programmatic roll-out: an observational and case-control study. *Lancet Infect Dis* **2015**, *15*, 422-428, doi:10.1016/S1473-3099(14)71060-6.
131. Simonsen, L.; Morens, D.; Elixhauser, A.; Gerber, M.; Van Raden, M.; Blackwelder, W. Effect of rotavirus vaccination programme on trends in admission of infants to hospital for intussusception. *Lancet* **2001**, *358*, 1224-1229, doi:10.1016/S0140-6736(01)06346-2.
132. Verstraeten, T.; Baughman, A.L.; Cadwell, B.; Zanardi, L.; Haber, P.; Chen, R.T.; the Vaccine Adverse Event Reporting System, T. Enhancing Vaccine Safety Surveillance: A Capture-Recapture Analysis of Intussusception after Rotavirus Vaccination. *American Journal of Epidemiology* **2001**, *154*, 1006-1012, doi:10.1093/aje/154.11.1006.
133. Murphy, T.V.; Gargiullo, P.M.; Massoudi, M.S.; Nelson, D.B.; Jumaan, A.O.; Okoro, C.A.; Zanardi, L.R.; Setia, S.; Fair, E.; LeBaron, C.W., et al. Intussusception among infants given an oral rotavirus vaccine. *N Engl J Med* **2001**, *344*, 564-572, doi:10.1056/NEJM200102223440804.
134. Yih, W.K.; Lieu, T.A.; Kulldorff, M.; Martin, D.; McMahill-Walraven, C.N.; Platt, R.; Selvam, N.; Selvan, M.; Lee, G.M.; Nguyen, M. Intussusception Risk after Rotavirus Vaccination in US Infants. *New England Journal of Medicine* **2014**, *370*, 503-512, doi:10.1056/NEJMoa1303164.
135. Weintraub, E.S.; Baggs, J.; Duffy, J.; Vellozzi, C.; Belongia, E.A.; Irving, S.; Klein, N.P.; Glanz, J.M.; Jacobsen, S.J.; Naleway, A., et al. Risk of intussusception after monovalent rotavirus vaccination. *N Engl J Med* **2014**, *370*, 513-519, doi:10.1056/NEJMoa1311738.

136. Buttery, J.P.; Danchin, M.H.; Lee, K.J.; Carlin, J.B.; McIntyre, P.B.; Elliott, E.J.; Booy, R.; Bines, J.E.; Group, P.A.S. Intussusception following rotavirus vaccine administration: post-marketing surveillance in the National Immunization Program in Australia. *Vaccine* **2011**, *29*, 3061-3066, doi:10.1016/j.vaccine.2011.01.088.
137. Carlin, J.B.; Macartney, K.K.; Lee, K.J.; Quinn, H.E.; Buttery, J.; Lopert, R.; Bines, J.; McIntyre, P.B. Intussusception risk and disease prevention associated with rotavirus vaccines in Australia's National Immunization Program. *Clin Infect Dis* **2013**, *57*, 1427-1434, doi:10.1093/cid/cit520.
138. Patel, M.M.; López-Collada, V.R.; Bulhões, M.M.; De Oliveira, L.H.; Márquez, A.B.; Flannery, B.; Esparza-Aguilar, M.; Montenegro Renoiner, E.I.; Luna-Cruz, M.E.; Sato, H.K., et al. Intussusception Risk and Health Benefits of Rotavirus Vaccination in Mexico and Brazil. *New England Journal of Medicine* **2011**, *364*, 2283-2292, doi:10.1056/NEJMoa1012952.
139. Velazquez, F.R.; Colindres, R.E.; Grajales, C.; Hernandez, M.T.; Mercadillo, M.G.; Torres, F.J.; Cervantes-Apolinar, M.; DeAntonio-Suarez, R.; Ortega-Barria, E.; Blum, M., et al. Postmarketing surveillance of intussusception following mass introduction of the attenuated human rotavirus vaccine in Mexico. *Pediatr Infect Dis J* **2012**, *31*, 736-744, doi:10.1097/INF.0b013e318253add3.
140. Stowe, J.; Andrews, N.; Ladhani, S.; Miller, E. The risk of intussusception following monovalent rotavirus vaccination in England: A self-controlled case-series evaluation. *Vaccine* **2016**, *34*, 3684-3689, doi:10.1016/j.vaccine.2016.04.050.
141. Yung, C.-F.; Chan, S.P.; Soh, S.; Tan, A.; Thoon, K.C. Intussusception and Monovalent Rotavirus Vaccination in Singapore: Self-Controlled Case Series and Risk-Benefit Study. *The Journal of Pediatrics* **2015**, *167*, 163-168.e161, doi:10.1016/j.jpeds.2015.03.038.
142. Shui, I.M.; Baggs, J.; Patel, M.; Parashar, U.D.; Rett, M.; Belongia, E.A.; Hambidge, S.J.; Glanz, J.M.; Klein, N.P.; Weintraub, E. Risk of Intussusception Following Administration of a Pentavalent Rotavirus Vaccine in US Infants. *JAMA* **2012**, *307*, 598-604, doi:10.1001/jama.2012.97.
143. Loughlin, J.; Mast, T.C.; Doherty, M.C.; Wang, F.T.; Wong, J.; Seeger, J.D. Postmarketing Evaluation of the Short-term Safety of the Pentavalent Rotavirus Vaccine. *The Pediatric Infectious Disease Journal* **2012**, *31*.
144. Zaman, K.; Dang, D.A.; Victor, J.C.; Shin, S.; Yunus, M.; Dallas, M.J.; Podder, G.; Vu, D.T.; Le, T.P.; Luby, S.P., et al. Efficacy of pentavalent rotavirus vaccine against severe rotavirus gastroenteritis in infants in developing countries in Asia: a randomised, double-blind, placebo-controlled trial. *Lancet* **2010**, *376*, 615-623, doi:10.1016/S0140-6736(10)60755-6.
145. Madhi, S.A.; Cunliffe, N.A.; Steele, D.; Witte, D.; Kirsten, M.; Louw, C.; Ngwira, B.; Victor, J.C.; Gillard, P.H.; Cheuvart, B.B., et al. Effect of human rotavirus vaccine on severe diarrhea in African infants. *N Engl J Med* **2010**, *362*, 289-298, doi:10.1056/NEJMoa0904797.
146. Steele, A.D.; Neuzil, K.M.; Cunliffe, N.A.; Madhi, S.A.; Bos, P.; Ngwira, B.; Witte, D.; Todd, S.; Louw, C.; Kirsten, M., et al. Human rotavirus vaccine Rotarix provides protection against diverse circulating rotavirus strains in African infants: a randomized controlled trial. *BMC Infect Dis* **2012**, *12*, 213, doi:10.1186/1471-2334-12-213.
147. Gurgel, R.G.; Bohland, A.K.; Vieira, S.C.; Oliveira, D.M.; Fontes, P.B.; Barros, V.F.; Ramos, M.F.; Dove, W.; Nakagomi, T.; Nakagomi, O., et al. Incidence of rotavirus and all-cause diarrhea in northeast Brazil following the introduction of a national vaccination program. *Gastroenterology* **2009**, *137*, 1970-1975, doi:10.1053/j.gastro.2009.07.046.
